# The phytochrome-interacting factor PIL13 enhances water use efficiency under fluctuating light and drought resilience in rice and soybean

**DOI:** 10.1038/s42003-025-08605-8

**Published:** 2025-08-26

**Authors:** Chunmei Luo, Zhan Xu, Zubair Iqbal, Yanjun Li, Jemaa Essemine, Suyang Fang, Na Li, Kai Huang, Xiangshen Meng, Zhibin Li, Genyun Chen, Mingnan Qu

**Affiliations:** 1 Jiangsu Key Laboratory of Crop Genomics and Molecular Breeding, College of Agriculture, Yangzhou Yangzhou University, Yangzhou, China; 2 Yazhouwan National Laboratory, Sanya, China; 3 State Key Laboratory of Plant Molecular Genetics, CAS Center for Excellence in Molecular Plant Sciences, Shanghai, China; 4 Tianjin Agricultural University, Tianjin, China

**Keywords:** Abiotic, Plant physiology

## Abstract

In natural environments, fluctuating light (FL) conditions significantly influence plant growth by modulating the balance between photosynthesis and water loss through stomata, quantified as the intrinsic water use efficiency under fluctuating light (iWUE_FL_). This effect is particularly pronounced under drought stress (FL-DS). To elucidate the genetic basis of stomatal responses to FL-DS, we analyzed iWUE_FL_ variations across 206 rice accessions and identified *OsPIL13*, a phytochrome-interacting factor, as a key gene associated with iWUE_FL_ through genome-wide association studies. Functional validation revealed that overexpressing *OsPIL13* in rice (WYG7) and its homolog in soybean (DN50) enhanced iWUE_FL_ by 13% and 15%, respectively, under FL-DS, demonstrating its conserved role across species. Conversely, knockout of *OsPIL13* or mutation of a causal SNP in its promoter reduced iWUE_FL_ by at least 10%. Mechanistically, *OsPIL13* regulates stomatal responses by repressing *OsSAL1*, a chloroplast retrograde signal regulator, and activating *OsNHX1*, a vacuolar sodium/proton antiporter. These findings highlight the potential of *PIL13* in improving crop resilience to FL-DS, particularly in maize-soybean intercropping systems.

## Abstract (web summary)

A study reports that a phytochrome-interacting factor confers drought resistance by regulating stomatal movement under fluctuating light conditions. These findings underscore the importance of considering light effects when breeding for drought tolerance.

## Introduction

Water scarcity, mostly exacerbated by climate change, rapid industrialization, and urbanization, poses a critical challenge to agricultural sustainability, particularly in regions with extensive rice cultivation^1^. It is predicted that by 2025, ~15–20 million hectares of irrigated rice in Asia are expected to face water scarcity^2^. Consequently, it becomes challenging for farmers to produce more rice per unit of land, given the limited water available, to meet the increasing food demand of the growing population. The persistent need to enhance rice production per unit of land amid limited water resources underscores the urgency of developing new rice cultivars with improved water use efficiency (WUE)^3^.

Intrinsic WUE (iWUE) is a critical parameter in quantifying carbon uptake and water loss at leaf-to-continental scales^4^, reflecting the trade-off between photosynthetic carbon gain and water loss through stomatal conductance. It is defined as the ratio of photosynthetic rates (*A*) to stomatal conductance (*g*_s_). There are substantial genetic variations in iWUE among different species^5^, even within the same species, such as rice^6^, wheat, poplar, and cottonwood^7–9^. Earlier, iWUE has been considered a screening target for drought stress (DS) breeding in various studies^7,8,10^. However, their utility remains debated, as their correlation with drought tolerance is weak or inconsistent, mainly due to lack of consideration of environmental changes^8^. Therefore, identifying the new trait representing drought tolerance level under changing environments is essential to guide molecular selection for DS breeding^11,12^.

Fluctuating light (FL) is a significant environmental factor that influences iWUE through stomatal regulation, especially for the bottom leaves within a canopy^9^. Predominantly, FL usually occurs concomitantly with DS. The latter (DS) significantly delayed the induction kinetics of stomatal and mesophyll conductances after transitioning from low to high light conditions^13^. Thus, this delayed photosynthetic induction under FL further aggravated sensitive performance under DS. This evidence suggests that some common mechanisms regulate plant responses to both environmental events (FL and DS). However, the FL and DS combined stress (FL-DS) regulatory mechanism remains unclear, which needs to be deeply investigated and well interpreted.

GWAS has become a valuable tool for gene mining; however, research on the gene regulation of ecophysiological traits under combined stress using GWAS is limited^14^. In the current study, we aimed to explore iWUE traits with high genetic variation and heritability that could be used as a selection target for molecular-assisted breeding. In this regard, we investigated the natural variation of iWUE dynamics during FL-DS in both field and growth chamber using a Minicore rice population. The population comprises 206 accessions, known for its suitable population size and genetic diversity^15^ for exploring genes associated with photosynthetic and nitrogen (N) utilization traits^16,17^.

Through large-scale phenotyping, we found that iWUE_FL_ possessed high SNP heritability and a strong correlation with FL-DS tolerance. Based on GWAS, we observed a strong association between the allelic variation of a phytochrome-interacting factor (*OsPIL13*) and iWUE_FL_, which mainly attributes the variation to a v3 SNP at the *OsPIL13* promoter. A functional analysis suggests that overexpressing *OsPIL13* increases iWUE_FL_ by 13% during FL-DS. Importantly, the promotive effects of *PIL13* in iWUE_FL_ were also true in soybean, suggesting a conserved function of *PIL13* in both species. Transcriptome analysis revealed that two genes (*OsSAL1* and *OsNHX1*) carrying G-box binding motifs exhibited the most significantly contrasting expressions. The binding ability and transcriptional regulation by *OsPIL13* upon the *OsSAL1* and *OsNHX1* were further confirmed by a yeast one-hybrid (Y1H) assay and chromatin immunoprecipitation (ChIP)-qPCR. This investigation sheds light on the genetic mechanisms underlying *OsPIL13*-regulated iWUE dynamics through coordination with *OsSAL1* and *OsNHX1* during FL-DS in rice. By unraveling *OsPIL13* elite variation and the intricate interactions, our study provides valuable insights to guide molecular selection breeding strategies aimed at improving rice grain production and water conservation, which could also work in other species, including soybean in maize-soybean intercropping practices.

## Results

### iWUE_FL_ as a crucial indicator for tolerance to FL-DS

**Fig. 1:**
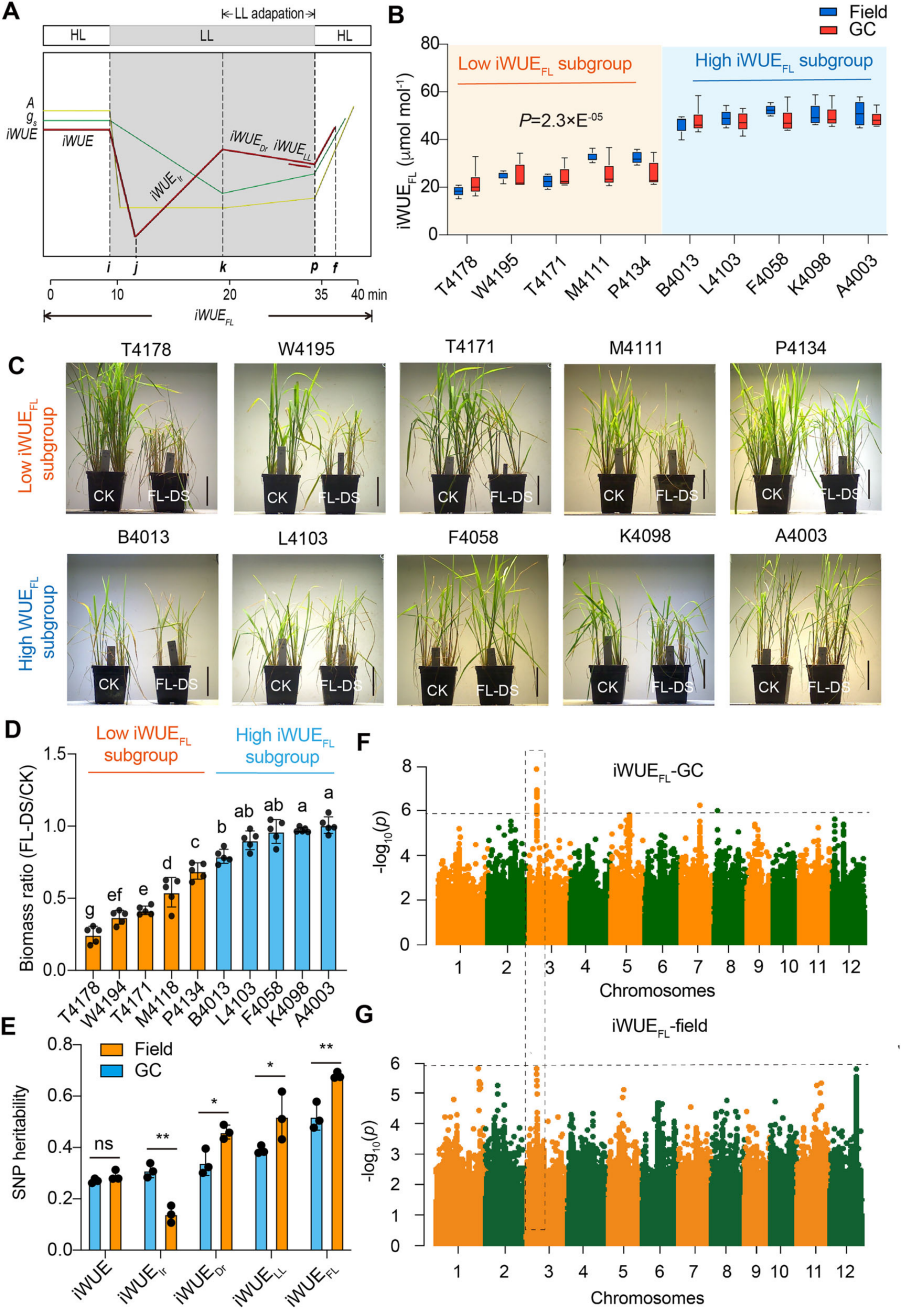
Determining candidate genes underlying the intrinsic water use efficiency during fluctuating light (iWUE_FL_) in rice Minicore population by genome-wide association study (GWAS). **A** A diagram displaying the definition of each iWUE-related trait during the FL condition. **B** Comparison of iWUE_FL_ in ten rice accessions with enormously differed iWUE_FL_ from the Minicore population grown under field and GC exposed to FL-DS. GC growth chamber. *n* = 4 (individual plants). **C** Imaging of ten rice accessions grown in a GC exposed to a 20d FL-DS. The vertical bar represents 10 cm. **D** Biomass ratio in ten rice accessions grown in GC with FL-DS relative to CK. Data are mean ±s.d. (*n* = 5 individual plants). **E** SNP heritability in five iWUE parameters in rice Minicore population grown under field and GC conditions. **F**, **G** Manhattan plots for iWUE_FL_ of rice accessions grown under field and GC conditions. The overlapping genomic region surrounding the peaked SNP associated with iWUE_FL_ between field and GC conditions was highlighted by the vertical dashed box.

To explore which iWUE parameters possess high heritability and correlate with tolerance to FL-DS, we investigated the natural variation of four iWUE-related traits, including iWUE_Ir,_ iWUE_Dr_, iWUE_LL,_ and iWUE_FL_ during FL using 206 rice accessions in a Minicore population^18^. The four iWUE-related traits exhibited natural variation and a normal distribution in the Minicore population (Fig. 1A; Supplementary Figs. 1A–E, 2A–E; Supplementary Data 1). Among them, a parameter representing a balance index between photosynthesis and water loss via stomata during FL (iWUE_FL_), exhibited the highest positive correlation with the biomass ratio in FL-DS compared to the CK condition (*R* = 0.72) (Supplementary Fig. 3A–E; Supplementary Data 2). In particular, ten rice accessions (five with extremely high iWUE_FL_ and five with extremely low iWUE_FL_) maintained a similar iWUE_FL_ ranking between the field and GC conditions (Fig. 1B). Consistently, accessions with low iWUE_FL_ values exhibited severe growth impairment under FL-DS. Those accessions with high iWUE_FL_ values displayed relatively greater FL-DS tolerance, as reflected by the biomass ratio in FL-DS compared to the CK condition (Fig. 1C, D). Importantly, iWUE_FL_ showed a robust positive correlation between field and GC conditions (Supplementary Fig. 3F), suggesting high natural variation and low environmental plasticity of iWUE_FL_.

The heritability (*h*^2^_SNP_) calculations for each iWUE parameter aimed to evaluate genetic control^19^. The results indicated *h*^2^_SNP_ values > 0.26 (*P* < 0.05) across all iWUE parameters, with iWUE_FL_ exhibiting the highest *h*^2^_SNP_ value of 0.51 compared to the other three parameters under both field and GC conditions (Fig. 1E). This evidence suggests that iWUE_FL_ has high heritability and could be considered an important parameter for reflecting FL-DS tolerance (Supplementary Note1).

### Screening candidate genes responsible for the iWUE_FL_ trait

**Fig. 2:**
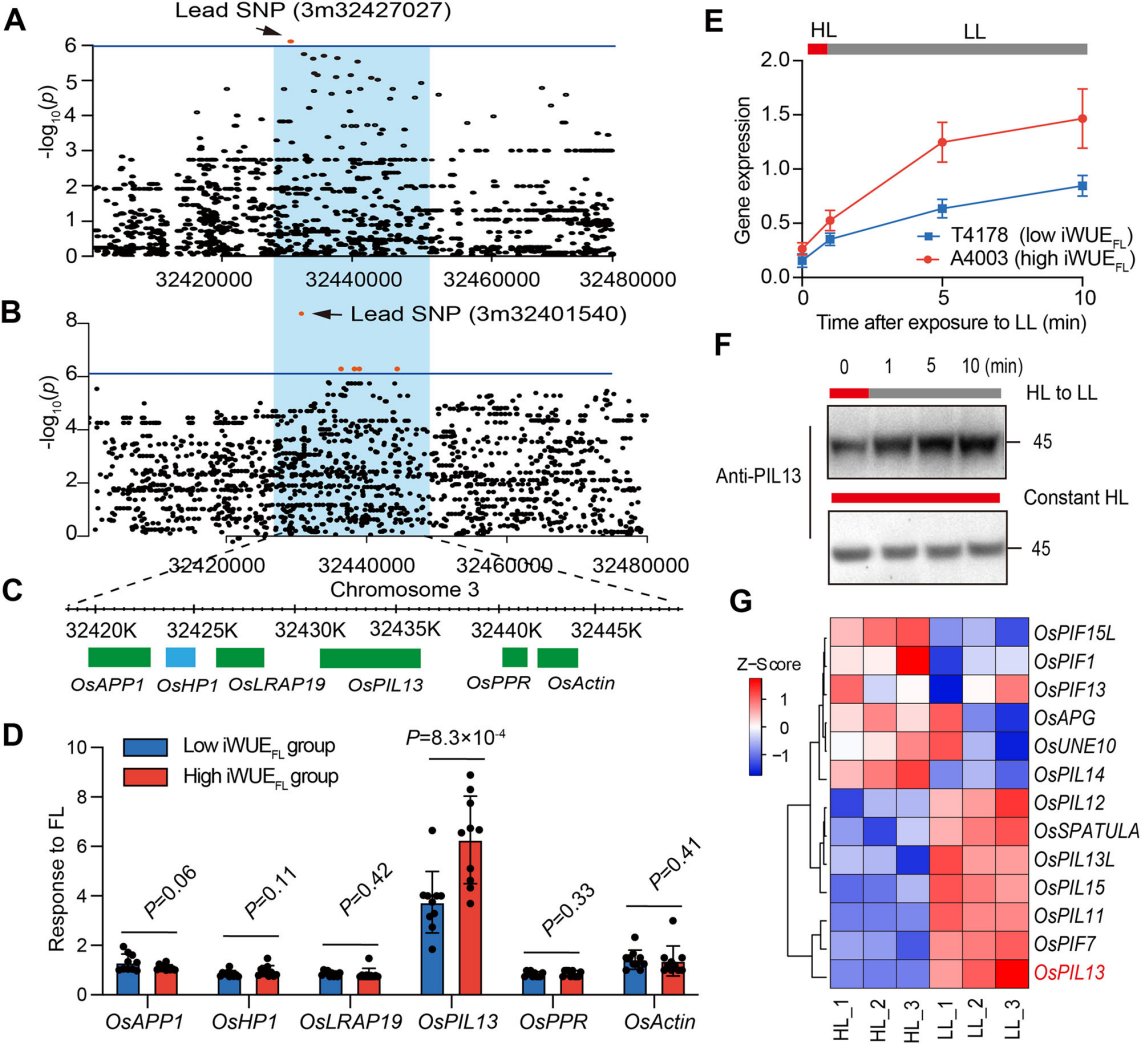
Identification of candidate genes underlying iWUE_FL_ through GWAS. Zoom-in Manhattan plots of iWUE_FL_ for field (**A**) and GC (**B**) conditions. The SNPs in the *OsPIL13* gene above the threshold of significant association were highlighted in red scatters. **C** List of six candidate genes that are located within the linkage disequilibrium (LD) block and their genomic positions. **D** Response of candidate genes, within the LD block surrounding the lead SNP, to FL-DS in two groups possessing contrasting iWUE_FL_ values. Plants were exposed to HL for 30 min, followed by 20 min LL, for qPCR experiments. Each group has 10 rice accessions in the Minicore population. Data are mean ± s.d. (*n* = 3 independent replicates). Student’s *t*-test was used to determine the significance level of the LL response of each gene between the two iWUE_FL_ subgroups. **E** *OsPIL13* gene expression levels followed by HL to LL transition treatment within 10 min between A4003 and T4178. Data are mean ± s.d. (*n* = 3 independent replicates). **F** Protein expression of *Os*PIL13 following HL to LL transition within 10 min in leaves of WYG7. **G** Heatmap representing the relative gene expression of 13 orthologous genes of *PIL13* in the rice genome in leaves of WYG7 exposed to either HL or LL conditions. The values for each cell are derived from three biological replicates.

Further explorations of candidate genes governing the iWUE_FL_ parameter, as well as the other three iWUE parameters (iWUE_Ir,_ iWUE_Dr_, and iWUE_LL_), were conducted using GWAS with a low-coverage genotype dataset (Fig. 1F, G; Supplementary Fig. 4A–D; Supplementary Note 2). Several known QTLs were identified in the genomic window of overlapped SNPs that were associated with these iWUE parameters, including *PsbP*^20^, *RM5521*^21^, *OsCML4*^22,23^, *Psb28*^24^, and *qD*_*ada*_*12*^25^ (Supplementary Fig. 4A–D). The Manhattan plots revealed a lead SNP (3m32427027, *p* value = 4.67 ×10^−06^) that was strongly associated with iWUE_FL_ in the field, while a lead SNP (3m32401540, *p* value = 8.12 ×10^−07^) was strongly related to iWUE_FL_ in GC (Fig. 2A, B). The two lead SNPs accounted for approximately 20% of the phenotypic variation (Fig. 1F, G). Notably, the two lead SNPs were located within the same linkage disequilibrium (LD) block window (32.42–32.45 Mb), where six candidate genes were identified (Fig. 2C).

To screen the candidate genes that are responsible for iWUE_FL_, we investigated the relative gene expression levels of these six candidate genes under 30 min of HL followed by 20 min of LL conditions (HL-LL transition treatment) using 20 rice accessions, with 10 belonging to the subgroup with an extremely high iWUE_FL_ (high iWUE_FL_ subgroup) and 10 belonging to the subset with an extremely low iWUE_FL_ (low iWUE_FL_ subgroup), and all plants were exposed to FL-DS (Supplementary Table 2). Interestingly, among the six candidate genes, only *OsPIL13* gene expression showed significant differences in response to LL between the high iWUE_FL_ and low iWUE_FL_ groups (*p* < 0.01) (Fig. 2D). Consistently, the expression level of *OsPIL13* in A4003, a *japonica* rice line from the Minicore panel with the highest iWUE_FL,_ was dramatically enhanced up to 5 fold higher than in T4178, an *indica* rice type from Minicore panel with the lowest iWUE_FL_ in Minicore population subjected to HL-LL transition treatment (Figs. 1B and  2E; Supplementary Table 2). The *Os*PIL13 protein contents were gradually increased following HL-LL transition treatment (Fig. 2F). These results reveal that *OsPIL13* is a potential candidate gene responsible for variation in iWUE_FL_ in the rice population.

### Spatiotemporal gene expression pattern of *Os*PIL13

According to the Rice Genome Annotation Database, the *OsPIL13* gene is annotated as a phytochrome-interacting factor (http://rice.uga.edu/). *Os*PIL13 is predicted to exhibit nuclear localization by RSLPred (http://www.imtech.res.in/ raghava/rslpred/), which was confirmed by the transient transformation in tobacco leaves (Supplementary Fig. 5A). Phylogenetic tree analysis revealed that *OsPIL13* displays weak sequence similarities with other 13 phytochrome-interacting factor genes in the rice genome (Supplementary Fig. 5B). Remarkably, among these phytochrome-interacting factor genes, the *OsPIL13* gene shows the most significant increase in its expression level following HL to LL transition treatment (Fig. 2G). The *OsPIL13* gene was predominantly expressed in leaves, followed by stem and root (Supplementary Fig. 5C). The expression level of *OsPIL13* was increased gradually from 5 to 60 DAG and declined at 80 DAG (Supplementary Fig. 5D). In comparison, the expression levels over five time-points within 80 DAG of *OsPIL13* in A4003, with the highest iWUE_FL_ in the Minicore population, was all higher than that in T4178, with the lowest iWUE_FL_ in the Minicore population (Supplementary Fig. 5D). Interestingly, the expression of *OsPIL13* gene was also stimulated by ABA treatment and exhibited a circadian rhythm pattern with low expression from 8:00 to 16:00, while it maintains a high expression from 20:00 to 04:00 as reported earlier in its homolog in Arabidopsis^26^ (Supplementary Fig. 6A, B).

### *OsPIL13* highly expressed under low light, particularly in the HapII subgroup

**Fig. 3:**
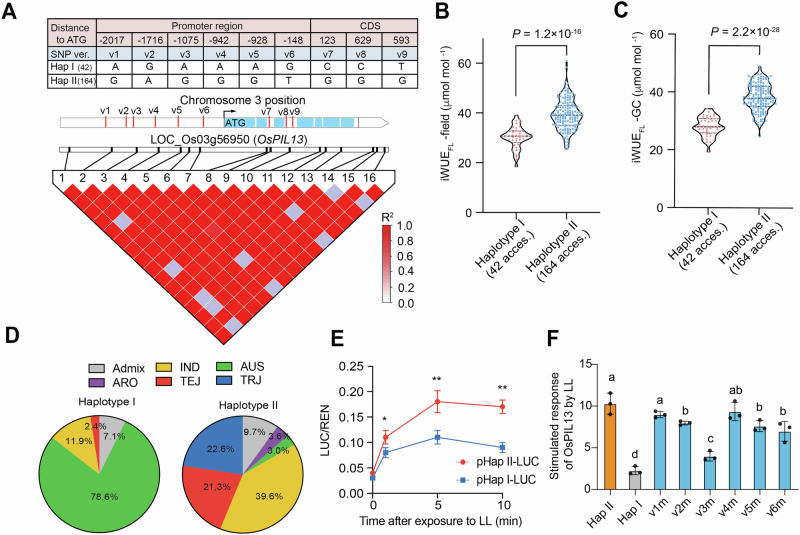
Haplotype analysis of *OsPIL13.* **A** Structure and haplotype analysis of *OsPIL13*. Distribution of iWUE_FL_ in the field (**B**) and in GC (**C**) in two haplotypes of the *OsPIL13* gene. The dotted lines represent the 0.25 and 0.75 quantiles, with median values indicated by bold lines. Differences between haplotypes were statistically analyzed using Student’s *t* test. The number of plants carrying Hap I and Hap II is shown in brackets. **D** Subpopulation distribution in two haplotypes of the *OsPIL13* gene. **E** Response of *OsPIL13* promoter activity to light during the switch from HL to LL within 10 min. Hap II and Hap I represent the two versions present in the *OsPIL13* promoter. Protoplast was exposed to HL for 30 min, followed by 20 min of LL. **F** Stimulated expression of *OsPIL13* by LL with different variations in the *OsPIL13* promoter. Values were calculated using the ratio of luciferase activities at 5 min of LL exposure against 0 min LL for each SNP version. The symbol “v(x)ml” represents six mutation versions introduced from Hap II into Hap I of the *OsPIL13* promoter. For panels (**E, F**), data are mean ± s.d. (*n* = 3 independent replicates). For panel (**E**), the symbols “*”, “**” and “***” were used to represent the significant differences for each time point at *P* < 0.05, *P* < 0.01, and *P* < 0.001, respectively, based on Student’s *t* test. For panel (**F**), different le*t*ters indicate significant differences based on one-way *ANOVA* followed by Tukey’s HSD tests (*P* < 0.05).

To fully resolve the DNA sequence variation in *OsPIL13* that might have been missed by low-coverage genome sequencing^27^, we re-sequenced *OsPIL13* in the Minicore population and identified 46 SNPs and 6 InDels in *OsPIL13* (Supplementary Data 3; Supplementary Note 3). Through GWAS screening of these 52 variants, nine SNPs were detected that were strongly associated with the variation of iWUE_FL_ under both field and GC conditions (*P* value < 1.87 × 10^−6^) (Supplementary Fig. 7; Supplementary Tables 3, 4). These nine SNPs of *OsPIL13* are integrated in a single LD block, and among them, six SNPs were positioned in the promoter and three within the intron regions of the *OsPIL13* gene (Fig. 3A; Supplementary Table 4). This evidence suggests that variation in iWUE_FL_ could be associated with the expression levels of *OsPIL13* rather than protein structure variation.

The nine SNPs were divided into two haplotypes, named Haplotype I (Hap I) and Haplotype II (Hap II), which comprise 42 and 164 accessions, respectively (Fig. 3A; Supplementary Table 4). Among these accessions, plants with Hap II exhibited significantly higher iWUE_FL_ values than those in Hap I in both field and GC conditions (Fig. 3B, C). Consistently, of the ten rice accessions that were screened based on their contrasting iWUE_FL_ values, the five with low iWUE_FL_ values all belong to Hap I, while those with high iWUE_FL_ values belong entirely to Hap II (Fig. 1B; Supplementary Table 2). Additionally, almost 90% of rice accessions in Hap I were of the *indica*-type (including IND and AUS). In contrast, nearly half of the rice accessions of Hap II were *japonica* type (including TEJ, TRJ, and ARO) (Fig. 3D), suggesting that the *japonica* subgroup preferentially carries the elite *OsPIL13* haplotype (Hap II).

Furthermore, *OsPIL13* promoter activity was more strongly stimulated in the Hap II promoter (amplified from A4003) than in the Hap I promoter (amplified from T4178) during the HL to LL transition within 10 min in rice protoplasts (*p* < 0.01) (Fig. 3E). Moreover, we assessed the effects of six SNPs at *OsPIL13* promoter region during HL-LL transition through luciferase-based promoter activity approach. This was achieved by introducing different SNPs from Hap II into the Hap I promoter. The findings indicate that the third SNP (v3), positioned at −1075 bp from the start codon of the *OsPIL13* gene, exhibited the lowest sensitivity to HL-LL transition treatment (Fig. 3F).

### *OsPIL13* gene function validation in vivo using transgenic techniques

To validate the function of the *OsPIL13* gene, we created an *OsPIL13* mutant (*ospil13*) at its third exon using CRISPR/CAS9, resulting in the loss of function of *OsPIL13* in WYG7. *ospil13* shows greater sensitivity to FL-DS with abolished *Os*PIL13 protein compared to WYG7 (Supplementary Fig. 8A, B). This led to a 26%, 20%, and 11% reduction in iWUE_FL_, tiller number, and yield in *ospil13* compared to WYG7, respectively, after an exposure to 20 d FL-DS treatment (Supplementary Fig. 8C–E).

**Fig. 4:**
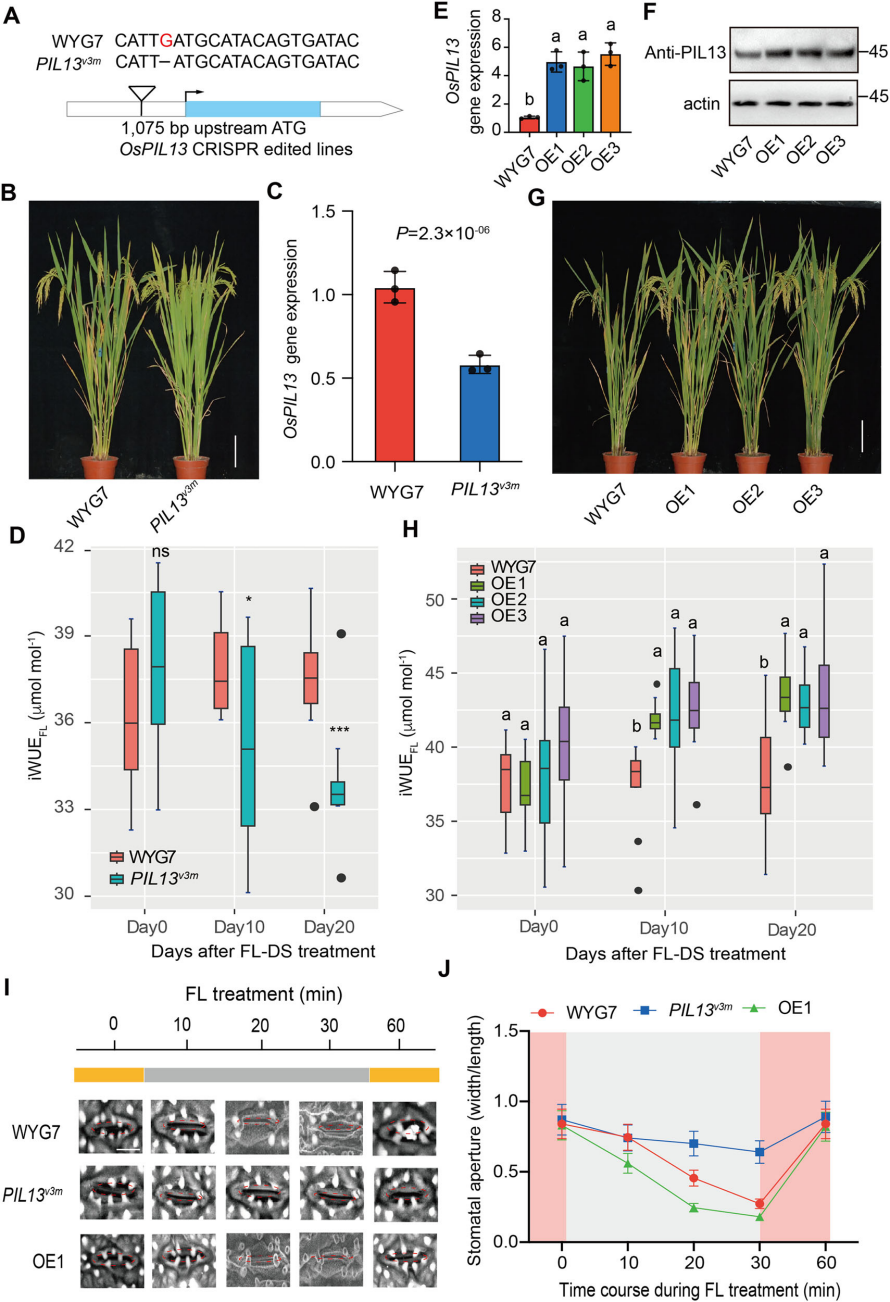
*OsPIL13* regulates iWUE and iWUE_FL_ under a combined FL-DS condition. **A** A nucleotide “G” at v3 SNP was deleted by CRISPR/CAS9 in the background of the WYG7 rice line (*PIL13*^*v3m*^). **B** Image of WYG7 and *PIL13*^*v3m*^ at the grain filling stage. The white vertical bar represents a scale of 10 cm. **C** Gene expression levels of *OsPIL13* in WYG7 and *PIL13*^*v3m*^ rice line. **D** iWUE_FL_ in WYG7 and *PIL13*^*v3m*^ rice line following different days of FL-DS treatment. **E** Gene expression levels of *OsPIL13* in WYG7 and *OsPIL13* overexpression (OE) lines. **F** Western blot showing *OsPIL13* expression in three OE lines. **G** Image of WYG7 and three *OsPIL13*-OE lines at their grain filling stage. The white vertical bar represents a scale of 10 cm. **H** iWUE_FL_ in WYG7, and three *OsPIL13*-OE rice lines following different days of FL-DS. **I**, **J** Stomatal aperture dynamics during FL. For panel (**I**), the shapes of stomatal apertures were marked in red circles. Bar, 10 μm. For panels (**C**, **D**), the symbols “*,” “**,” and “***” were used to represent the significant differences between FL-DS and CK at *P* < 0.05, 0.01, and 0.001, respectively, based on Student’s *t* test. ns non-significant. Each bar data represents the mean of n replicates ±s.d.: *n* = 3 (indepe*n*dent replicates) for panel (**C**) and *n* = 7 (individual plants) for panel (**D**). For panels (**E**, **H**), different letters represent significant differences among three *OsPIL13*-OE lines and WYG7 based on one-way *ANOVA* followed by Tukey’s HSD tests (*P* < 0.05). Each bar data represents the mean of n replicates ±s.d.: *n* = 3 (independent replicates) for panel (**E**) and *n* = 7 (individual plants) for panel (**H**).

To further examine the v3 SNP effects on iWUE_FL_, we created a knocking-down line of *OsPIL13* through CRISPR/CAS9, leading to the deletion of v3 SNP at the promoter, named *PIL13*^*v3m*^ (Fig. 4A). The result reveals that, like *ospil13* mutant, the performance of *PIL13*^*v3m*^ was impaired under FL-DS, compared to WYG7, together with 55% and at least 13% reduction in *OsPIL13* gene expression and iWUE_FL_, respectively (Fig. 4C, D). This led to a 16% increase in half-time of stomatal closure (τ_cl_) and to 20%, 25%, 15%, and 12% reduction in *A*, SPAD values (chlorophyll content), tiller number, and yield, respectively, in *PIL13*^*v3m*^ compared to WYG7 under FL-DS (Supplementary Fig. 9A–G).

In addition, to validate the Hap II effects of *OsPIL13*, we generated three overexpression (OE) lines of *OsPIL13* driven by its promoter from Hap II, leading to up to 3 times increase in *OsPIL13* gene expression together with around 2 times increase in *Os*PIL13 protein levels across OE lines (Fig. 4E, F). Plants of three *OsPIL13* OE lines show greater tolerance to FL-DS (Fig. 4G). Within 20d FL-DS treatment during the grain-filling stage, these OE lines exhibited at least a 13% increase in iWUE_FL_ compared to WYG7 (Fig. 4H). This leads to a 30% reduction in τ_cl_ and to increases of 20%, 7%, 20%, and 13% in *A*, SPAD records, tiller number, and yield, respectively, in three OE lines compared to WYG7 under FL-DS (Supplementary Fig. 10A–G).

Consistently, the stomatal aperture in *PIL13*^*v3m*^ shows slower closure than WYG7 during HL-LL transition treatment, which collaborates with the higher τ_cl_ compared to WYG7 (Fig. 4I, J; Supplementary Fig. 9A), and vice versa for *OsPIL13* OE lines (Fig. 4I, J; Supplementary Fig. 10A), suggesting that stomatal movement during FL-DS is strongly related to the changes in iWUE_FL_ regulated by *Os*PIL13.

Under CK condition, iWUE_FL_ values show a 7% decrease (*P* < 0.05) in *PIL13*^*v3m*^ and a 6% increase (*P* < 0.05) across three OE lines, compared to WYG7 (Supplementary Fig. 11A–C). However, no significant differences were observed in tiller numbers and yield per plant when *PIL13*^*v3m*^ and WYG7 or the three *OsPIL13*-OE lines were compared to WYG7 (Supplementary Fig. 11D–G). These results further emphasize that *Os*PIL13 regulates iWUE_FL,_ predominantly under FL-DS conditions.

### Greater regulation of *Os*PIL13 on iWUE_FL_ under FL-DS than under either FL or DS

Stomatal induction differs under combined FL-DS events compared to their single case^13^. To compare the effects of FL-DS with single cases (FL or DS) on iWUE_FL_, we treated plants using an OE line (OE1) with WYG7 in either the FL or DS condition. Results suggest that expression level of *OsPIL13* was higher in FL-DS than under either FL or DS condition (Supplementary Fig. 12A). Thus, a higher expression level of *OsPIL13* was observed under FL than under DS, leading to a 13% and 8% increase in iWUE_FL_ in OE line compared to WYG7 under FL and DS, respectively (Supplementary Fig. 12A–C). We also observed a 15% and 10% decrease in τ_cl_ in OE line compared to WYG7 under FL and DS, respectively (Supplementary Fig. 12D). A higher enhancement in both tiller number and yield in OE line compared to WYG7 was observed under FL than under DS (Supplementary Fig. 12E, F). This evidence further corroborates that FL-DS significantly affects iWUE_FL_ more than either FL or DS event.

### Screening downstream regulatory genes of *Os*PIL13

**Fig. 5:**
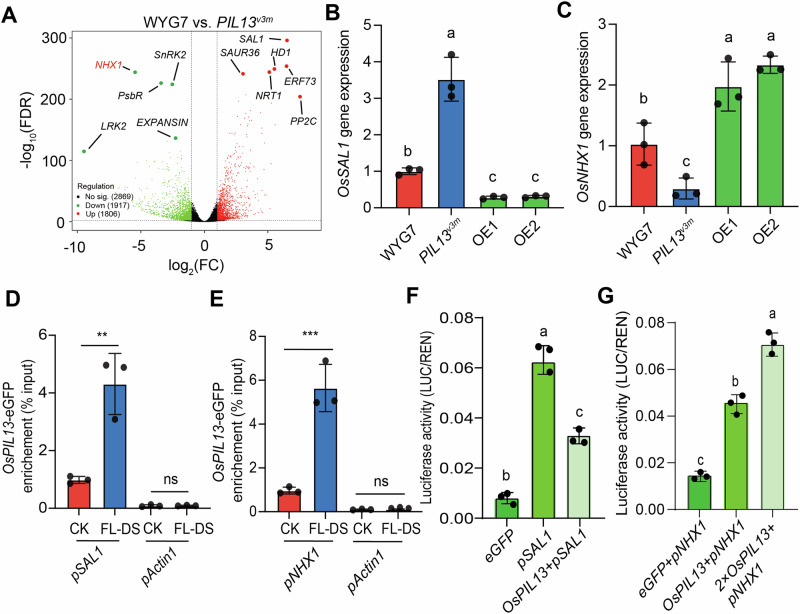
Identification of *OsPIL13* downstream genes based on transcriptome analysis. **A** Volcano plot representing differentially expressed genes (DEGs) between WYG7 and *PIL13*^*v3m*^ under FL-DS based on transcriptome analysis. The samples were collected from WYG7 and *PIL13*^*v3m*^ rice lines after an exposure to 20d FL-DS treatment. **B**, **C** *OsSAL1*, and *OsNHX1* gene expression levels in WYG7, *PIL13*^*v3m*^, and two *OsPIL13* OE rice lines under FL-DS. **D**, **E** ChIP-qPCR experiments for *Os*PIL13 regulating *OsSAL1* gene expression in *OsPIL13*-OE1 rice line subjected to either 20d of FL-DS or CK using eGFP beads for affinity. **F**, **G** Luciferase activity determination for *Os*PIL13 transcriptionally represses the expression of *OsSAL1*. For panels (**B**, **C**) and (**F**, **G**), different letters represent the significant differences based on one-way *ANOVA* followed by Tukey’s HSD tests (*P* < 0.05), while for panels (**D**, **E**), the symbol “**” was used to represent the significant differences between FL-DS and CK at a *P* < 0.01 based on Student’s *t* test. ns non-significant. *n* = 3 (independent replicates) for panels (**A**–**C**, **E**, **F**).

To further elucidate the molecular mechanism of *Os*PIL13 underlying the iWUE_FL_, we resorted to transcriptome analysis. The obtained results showed that the mean clean reads for both WYG7 and *PIL13*^*v3m*^ are 21,467,615, which maps to 92.5% of the raw reads, and the mean Q_30_ values are 0.94, based on the quality control of the transcriptome analysis (Supplementary Table 5; Supplementary Data 2). We identified 1,806 upregulated DEGs and 1,917 downregulated DEGs (Fig. 5B). In particular, we found that the *OsSAL1* gene was highly upregulated, and the *OsNHX1* gene was dramaticallly downregulated in the top 1% list of DEGs (Fig. 5B). Consistently, the increased expression of *OsSAL1* and the reduced expression of *OsNHX1* were measured in *PIL13*^*v3m*^ compared to WYG7 (Fig. 5B, C), the two genes show reversed expression patterns, as observed in *PIL13*^*v3m*^, in the two OE lines of *OsPIL13*, compared to WYG7 exposed to FL-DS (Fig. 5B, C).

ChIP-qPCR results show a dramatic fold enrichment in the promoters of both *OsSAL1* and *OsNHX1* binding with *Os*PIL13 in the *OsPIL13*-OE1 rice line exposed to 20d of FL-DS, compared to CK (Fig. 5D, E), which implies that *Os*PIL13 harbors strong affinity with *OsSAL1* and *OsNHX1* especially under FL-DS condition, as observed in Y1H assays (Supplementary Figs. 13, 14). Consistently, luciferase activity assay strongly suggested that *Os*PIL13 transcriptionally inhibits and activates the expression of the *OsSAL1* and *OsNHX1*, respectively (Fig. 5F, G).

### *Os*PIL13 regulates stomatal adjustment through *Os*SAL1 and *Os*NHX1

Both *Os*SAL1 and *Os*NHX1 were significantly involved in stomatal adjustment through different biological pathways. The SAL1-PAP retrograde pathway is known for interacting with ABA signaling, crucial in regulating stomatal closure and drought resistance^28^, while *Os*NHX1 represents a proton sodium exchanger responsible for stomatal dynamics during DS^16^. To further investigate the regulation of iWU_FL_ by *Os*PIL13 through the expression of *OsSAL1* and *OsNHX1*, we established OE rice lines for both *OsSAL1* and *OsNHX1* (*OsSAL1*-OE and *OsNHX1*-OE) individually driven by their native promoters, an *OsSAL1* mutant (*ossal1*), and co-expression rice lines of *OsPIL13*-*OsSAL1* and *OsPIL13*-*OsNHX1*.

**Fig. 6:**
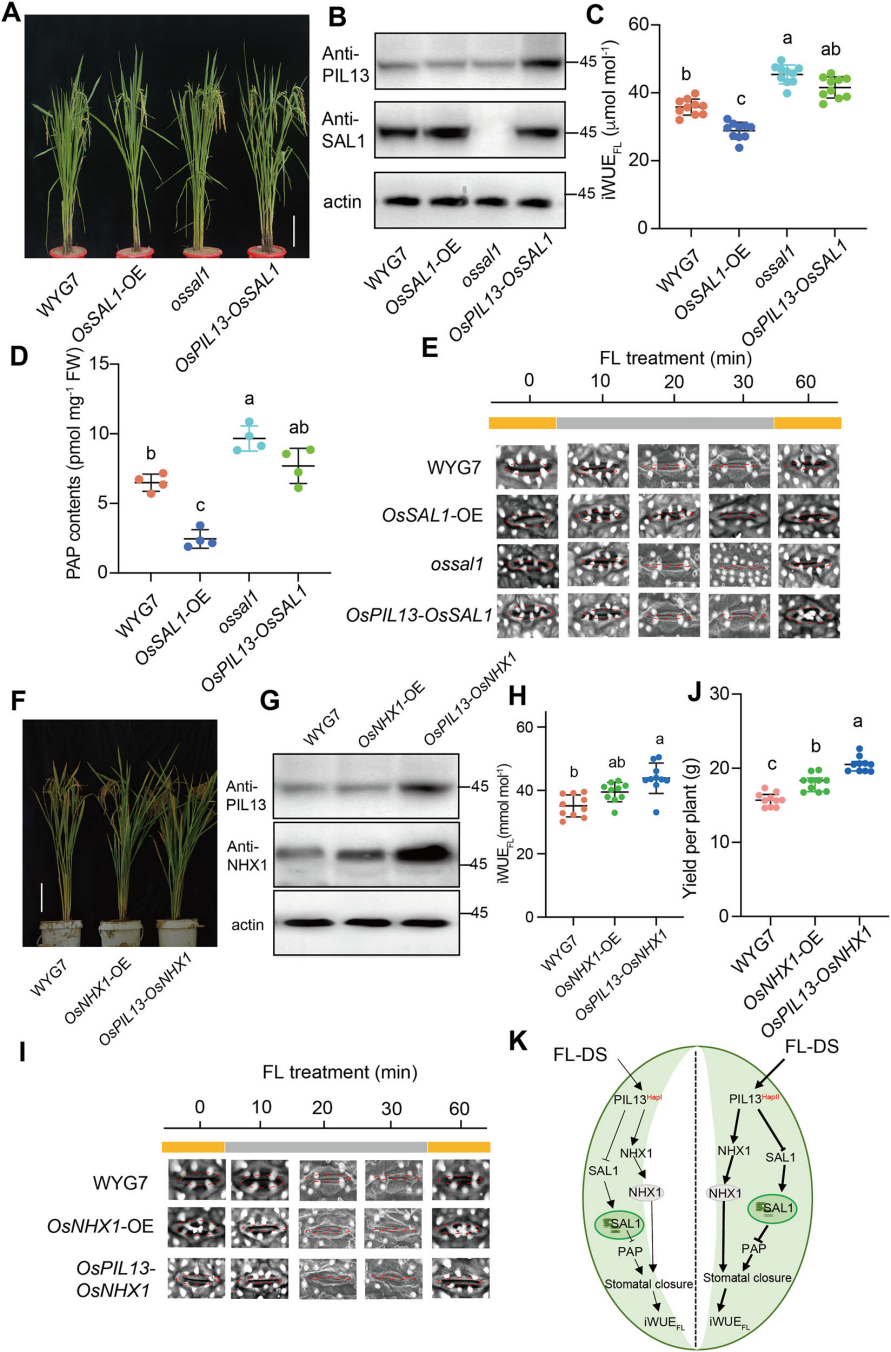
*Os*PIL13 directly regulates *OsSAL1* and *OsNHX1* gene expression, and enhances drought tolerance to FL-DS. **A** Images of WYG7, OE lines of *OsSAL1* (*OsSAL1*-OE), *ossal1* mutant, and co-overexpression of both *OsSAL1* and *OsPIL13* (*OsPIL13*-*OsSAL1*) exposed to 20d FL-DS at the graining stage. The vertical bar represents a scale of 10 cm. **B** Western blot showing OsSAL1 expression levels in WYG7, *OsSAL1-*OE*, ossal1*, and *OsPIL13-OsSAL1*. **C**, **D** iWUE_FL,_ and PAP contents in WYG7, *OsSAL1-*OE*, ossal1*, and *OsPIL13-OsSAL1*. Each bar represents the mean of n replicates (*n* = 10, individual plants) for panel (**C**) and (*n* = 4, individual plants) for panel (**D**). **E** Stomatal aperture dynamics in WYG7, *OsSAL1-*OE*, ossal1*, and *OsPIL13-SAL1* during FL treatment. The shapes of the stomatal apertures were marked in red circles. Bar, 10 μm. **F** Images of WYG7, *OsNHX1*-OE, and *OsPIL13*-*OsNHX1* exposed to 20d FL-DS at the graining stage and images of WYG7 and *OsPIL13*-*OsNHX1* exposed to 20d DS in the field. The vertical bar represents a scale of 10 cm. **G** Western blot showing OsNHX1 protein expression levels in WYG7, *OsNHX1-*OE, and *OsPIL13-OsNHX1* under FL-DS. **H**–**J** iWUE_FL,_ and yield per plant in WYG7, *OsNHX1-*OE, and *OsPIL13-OsNHX1* under FL-DS. Each bar of data represents the mean of n replicates (*n* = 10, individual plants) for panels (**H**–**J**). **I** Stomatal aperture dynamics in WYG7, *OsNHX1-*OE, and *OsPIL13-OsNHX1* under FL-DS. The shapes of the stomatal apertures were marked in red circles. Bar, 10 μm. **K** Summary model representing the regulation of *Os*PIL13 with two haplotypes on iWUE_FL_ under FL-DS through collaborating with *OsSAL1* and *OsNHX1*. For panels (**C**, **D**, **H**–**J**), different letters represent significant differences among groups based on one-way *ANOVA* followed by Tukey’s HSD test (*P* < 0.05).

Our findings revealed that *OsSAL1*-OE exhibited a significant impairment in growth under FL-DS conditions, simultaneous to a substantial increase in *Os*SAL1 protein levels relative to WYG7. In contrast, the *ossal1* mutant displayed better growth performance compared to WYG7 under FL-DS conditions (Fig. 6A, B). Although less pronounced than in *ossal1*, the growth of *OsPIL13*-*OsSAL1* was considerably better than that for both WYG7 and the *OsSAL1*-OE line, suggesting that *Os*PIL13 compensates for the *Os*SAL1 inhibitory effects under FL-DS conditions (Fig. 6A, B). Corresponding to their performance under FL-DS, iWUE_FL_ contributes to a 13% reduction in *OsSAL1*-OE and 15% increase in *ossal1*. In addition, *OsPIL13-OsSAL1* has similar enhanced effects with *ossal1* (Fig. 6C). As a downstream metabolic substrate of *Os*SAL1, PAP contents were conversely corresponding to the expression of *OsSAL1* in *OsSAL1*-OE, and *ospil13* under FL-DS (Fig. 6D). This corresponds to the rapidity of stomatal closure during HL-LL transition treatment among *OsSAL1*-OE, *ossal1*, and *OsPIL13-OsSAL1* (Fig. 6E; Supplementary Fig. 15A, B). In addition, tiller number and yield show corresponding changes as iWUE_FL_ was performed in these rice lines (Fig. 6C; Supplementary Fig. 15C, D). These findings confirm the regulation of stomatal adjustment by *Os*PIL13 through the *Os*SAL1-PAP retrograde pathway.

Next, we analyzed *Os*PIL13, which is expected to regulate an alternative pathway of stomatal adjustment through *OsNHX1*. Thus, both *OsNHX1*-OE and *OsPIL13*-*OsNHX1* rice lines exhibited better performance than WYG7 under FL-DS (Fig. 6F), concomitant with a gradual increase in *Os*NHX1 protein levels in *OsNHX1*-OE and *OsPIL13*-*OsNHX1* rice lines (Fig. 6G). Compared to WYG7, iWUE_FL_ values were increased by 11% and 19% in *OsNHX1*-OE and *OsPIL13*-*OsNHX1*, respectively (Fig. 6H), while decreased by 10% in *osnhx1* under FL-DS (Supplementary Fig. 16A, B). Both stomatal aperture and g_s_ dynamics data during the HL-LL transition show faster stomatal closure in *OsNHX1*-OE and *OsPIL13*-*OsNHX1* (Fig. 6I; Supplementary Fig. 16C, D). PAP contents were not significantly altered in *OsNHX1*-OE, while it was increased dramatically in *OsPIL13-OsNHX1* rice line (Supplementary Fig. 16E), confirming that cellular PAP consumption was due to *Os*SAL1 but not to *Os*NHX1. In addition, the changes of both tiller number and yield mirror the results of iWUE_FL_ in these *OsNHX1* overexpression lines (*OsNHX1*-OE and *OsPIL13*-*OsNHX1*) under FL-DS (Fig. 6J; Supplementary Fig. 16F).

Further exploration was performed to validate the regulation of *OsSAL1* and *OsNHX1* by *Os*PIL13 through in vivo transcriptional dynamics of these genes in response to FL-DS. As expected, results suggest that *OsNHX1* displays a similar expression pattern as *OsPIL13* does (Supplementary Fig. 17A). *OsSAL1* shows a reversed expression pattern with *OsPIL13* during FL-DS (Supplementary Fig. 17A), and the expression of *OsPIL13* exhibits more rapid response than those of the two other downstream genes (*OsSAL1* and *OsNHX1*). In addition, the expression levels of *OsNHX1* and *OsSAL1* were not changed in the mutants of their counterpart, while it is valid for the expression of the *OsPIL13* gene in either *osnhx1* or *ossal1* mutants under FL-DS (Supplementary Fig. 17B–E).

Under CK condition, *OsSAL1*-OE, *OsPIL13*-OE1, and *OsPIL13*-*OsSAL1* did not show significant differences in their performance, as well as tiller number and yield, compared to WYG7, despite iWUE_FL_ and PAP contents being significantly higher in *osal1* and *OsPIL13-OsSAL1* compared to *OsSAL1*-OE (Supplementary Fig. 18A–E). In addition, the performances of the *OsNHX1*-OE and *OsPIL13*-*OsNHX1* rice lines, as displayed by tiller number and yield, were not significantly altered in either *OsNHX1* or *OsPIL13*-*OsNHX1*, compared to WYG7, although iWUE_FL_ was increased dramatically in *OsNHX1* and *OsPIL13*-*OsNHX1*, the promotive effects were less pronounced than those under FL-DS condition (Fig. 6H; Supplementary Fig. 19A–D). These findings suggest that the effectiveness of *OsSAL1* and *OsNHX1* regulation by *Os*PIL13 on iWUE_FL_ was more pronounced under FL-DS than under CK condition.

We summarized that the FL-DS event triggered a marked increase in *OsPIL13* gene expression, which in turn induces an increase in *OsNHX1* expression, leading to accelerated stomatal closure and, consequently, enhanced water conservation, especially in *OsPIL13* Hap II rice types (Fig. 6K). Simultaneously, *Os*PIL13 transcriptionally represses *OsSAL1* expression, leading to the accumulation of PAP and rapid stomatal closure. These dual pathways regulated by *Os*PIL13, eventually promote iWUE_FL_ during FL and enhance tolerance to FL-DS (Fig. 6K).

### Local light regime shapes the haplotypic distribution of *OsPIL13*

Geographical and environmental gradients shape phenotypic trait variation and genetic structure in plants^29^. To understand the relationship between extreme FL-DS environments and *OsPIL13* haplotypic distribution, we analyzed the originated climate information for each rice accession in the Minicore population. We found that local light irradiance features, including annual total solar irradiance, direct total solar irradiance, and mean yearly visibility, were significantly lower in rice accessions with Hap I compared to Hap II. In contrast, mean precipitation was considerably higher in Hap I rice accessions than in Hap II ones, although there are no significant changes in the mean annual temperature (Supplementary Fig. 20A–E).

As mentioned above, *japonica* rice types preferentially exist in the Hap II group of *OsPIL13* (Fig. 3D). Compared to *indica*, it has been reported to have a higher WUE and is mainly grown in the temperate region^30,31^. Consistently, we found regional occurrences and distributions of the two *OsPIL13* haplotypes: Hap II is found primarily in North America, Southeast Asia, and Western Europe, with a dominant regional presence in China, Japan, and United States, whereas Hap I is preferentially present in the regions that typically experience extreme DS events from Africa, South America, Southeast Asia, and the Indian subcontinent, including Mali, Egypt, Iran, Argentina, Indonesia, India, and Pakistan. These observations indicate that the haplotypic distribution of *OsPIL13* is widely related to local light regimes and DS, where rice accessions were selected, and iWUE_FL_ experiences environmental pressure for FL-DS adaptation.

### Conserved function of *OsPIL13* in iWUE_FL_ in soybean

**Fig. 7:**
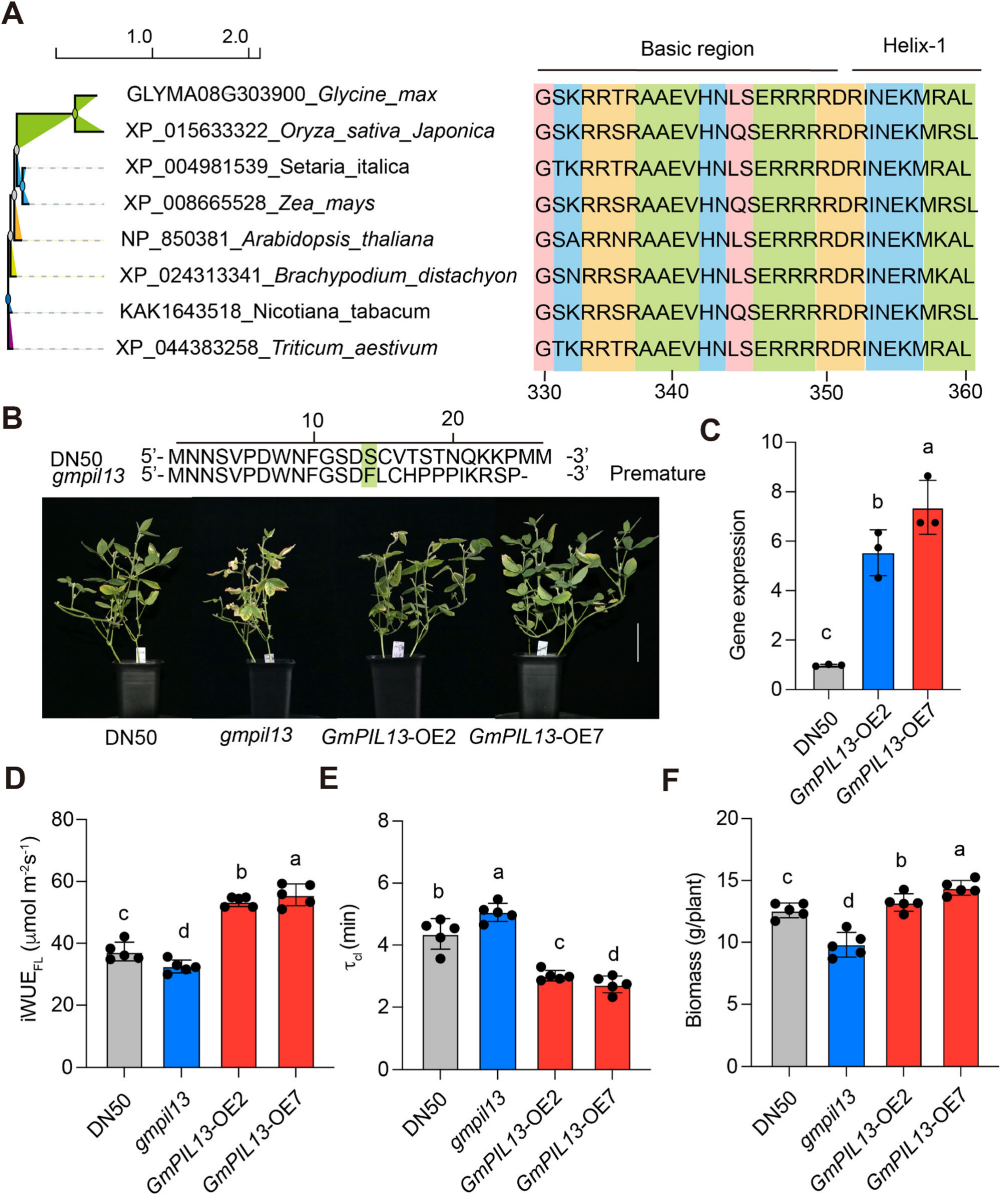
*Gm*PIL13 promotes iWUEFL and biomass accumulation under FLDS condition in *Glycine max*. **A**, Phylogenetic tree analysis on the amino acids of *Os*PIL13 homologs among different species. The domain architecture of *Os*PIL13 was annotated according to Gao et al. (2022)^17^. **B**, Performance of a CRISPR edited line (*gmpil13*) and two overexpression lines of *GmPIL13* in soybean (*Glyma.08G303900*) with the background of Dongnong 50 (DN50) under FL-DS for 20 d. 15 d old soybean seedlings in growth chamber with same growth condition as performed in rice were used for FL-DS treatments. The FL-DS treatments were conducted following same protocol as performed in rice in growth chamber. CRISPR editing brings a “G” insertion at 41-bp downstream of ATG leading to premature translation termination at 25^th^ amino acids of *Gm*PIL13. The vertical bar represents a scale of 10 cm. **C**, Gene expression levels of *GmPIL13* in two overexpression lines of *GmPIL13*. Relative expression levels were determined using *GmPIL13* against *Tublin* (reference gene). The primer was listed in Supplementary Table 1. **D-F**, iWUE_FL_, τ_cl_ and biomass in *gmpil1*3 and two overexpression lines of *GmPIL13* exposed to FL-DS. Each bar data represents the mean of n replicates (*n* = 3 individual plants for panel **C** and *n* = 5 individual plants for panels **D-F**) ±s.d. Different letters represent the significance level for each trait among DN50, *gmpil13* and two overexpression lines of *GmPIL13* based on Tukey’s HSD test (*P* < 0.05).

To explore the broader biological significance across diverse species, we performed multiple sequence alignment of *Os*PIL13 with its homologs from representative species and observed that the protein sequences of PIL13 were very conserved in its bHLH domain^26^ (Fig. 7A). Following FL-DS, we found that 15% increase in iWUE_FL_ in two overexpression lines of *GmPIL13* (*GmPIL13*-OEs), with at least 6 times increase in gene expression than its WT (DN50) (Fig. 7B, C). Accordingly, τ_cl_ shows a 15% reduction together with a 12% enhancement in biomass in two *GmPIL13*-OE than DN50 (Fig. 7E, F). Conversely, knocking-out *GmPIL13* leads to 8% reduction in iWUE_FL_, a 10% increase in τ_cl_, and a 10% reduction in biomass than DN50 under FL-DS (Fig. 7D–F).

In terms of CK condition, we observed a less enhancement in both growth performance in the two overexpression lines of *GmPIL13* compared it to that under FL-DS condition (Fig. 7B; Supplementary Fig. 21A). The *GmPIL13* gene expression levels were stimulated by less than 1.5 times in two OE lines of *GmPIL13* (Supplementary Fig. 21B). Overexpressing *GmPIL13* leads to around 10% increase in iWUE_FL_, 5% decrease in τ_cl_ and 7% increase in biomass accumulation under CK condition (Supplementary Fig. 21C–E). In contrast, knocking-out *GmPIL13* shows around 10% increase in iWUE_FL_, 10% reduction in τ_cl_, and 4% increase in biomass under CK condition (Supplementary Fig. 21C–E).

## Discussion

FL triggers an undesirable iWUE and further aggravates sensitive performance under DS through altering stomatal movements^13,32^. However, the molecular mechanism underlying the genes responsible for this response remains largely unknown. In this study, we identified an iWUE-related trait, iWUE_FL,_ strongly correlated with biomass ratio under FL-DS over CK condition, rather than biomass under either FL-DS or CK condition. We observed that a v3 SNP carrying the “G” allele from Hap II at the *OsPIL13* promoter conferred the tolerance to FL-DS through enhancing iWUE_FL_. We demonstrated that *Os*PIL13 directly regulated the expression of *OsSAL1* and *OsNHX1* and, hence, promoted the iWUE_FL_ during FL-DS. The new mechanism could help molecular breeding for rice to cope with such combined stress conditions.

First, performing an appropriate phenotyping approach and selecting a suitable natural population are two keys for identifying genes responsible for photosynthetic traits with high heritability used for GWAS. In this study, our phenotyping results show a robust correlation of iWUE_FL_ between field and GC experiments (Fig. 1B; Supplementary Fig. 3F), which confirms the effectiveness of the approach of transplanting plants from the field to GC as reported earlier in rice^33^. Previous studies have reported many genes that regulate photosynthetic efficiency, such as NRP1^34^, *EmBP1*^35^, and *PsbS*^36^. However, very few studies have documented the photosynthetic regulatory genes using GWAS. Thus, applying GWAS on the rice Minicore population collaborating with the phenotyping screening, we discovered 20 SNPs shared across various iWUE parameters. Notably, the candidate gene *RM5521* on chromosome 2 (Chr 2)^21^ appeared within the LD block (15.56–16.13 Mb) associated with iWUE_Ir_ and iWUE (Supplementary Fig. 4A, C). Furthermore, our investigation identified *OsCML4* (29.16–30.57 Mb) on chromosome 3 (Chr 3)^22,23^ within the candidate genes governing iWUE, iWUE_LL,_ and iWUE_FL_ (Supplementary Fig. 4A, D). These genes were previously reported to be related to the photosynthetic traits, suggesting a successful case of using the Minicore population for GWAS on the iWUE-related traits in our study. Therefore, the rice Minicore population was always used for GWAS on photosynthetic characteristics, as it has a proper population size and huge genetic diversity^15,20,37^.

We then employed GWAS using the Minicore population to identify the gene responsible for a novel trait, iWUE_FL_, with high SNP heritability and positive correlation to FL-DS performance. In this regard, we identified a strong association between allelic variations of *OsPIL13* with iWUE_FL_ and validated the promoted effects of *Os*PIL13 on iWUE_FL_ by *PIL13*^*v3m*^, an *OsPIL13* mutant, and three *OsPIL13*-OE lines under FL-DS (Fig. 4; Supplementary Figs. 8–11). *Os*PIL13, belonging to the phytochrome-interacting factors (PIFs) gene family, is also named *Os*PIL1^38,39^. The PIFs were renowned for playing diverse roles, including internode elongation, stomatal movements, and photomorphogenic and ABA responses in plants^39–44^. PIFs typically accumulate in darkness, promoting skotomorphogenesis, while exposure to light triggers their rapid phosphorylation and subsequent degradation via the activity of phyB and phyA in Arabidopsis^45,46^. We confirmed that *OsPIL13* in rice gradually accumulated following exposure to LL within 10 min, together with its gene expression levels (Fig. 2E, F). Notably, the enhanced expression of *OsPIL13* induced by LL was more pronounced than that of its 12 orthologue genes in the rice genome (Fig. 2G). This evidence suggests a scenario akin to PIF1, where activated phyB interacts with *OsPIL13*, thus prompting its phosphorylation and degradation under light irradiation^47^.

Disconnection between *A* and *g*_s_ during FL is a significant factor in the changes of iWUE, especially under FL-DS^32^. It has been reported that sluggish stomatal closure during the HL-LL transition could cause water loss^37^. In our study, we observed faster stomatal closure speed as displayed by lower values of τ_cl_ and faster decline in stomatal aperture in *OsPIL13*-OE lines, relative to WYG7 (Fig. 4H, J; Supplementary Fig. 10A), and vice versa for *PIL13*^*v3m*^ (Fig. 4D, I, J; Supplementary Fig. 9A). This evidence reveals that stomatal movement and aperture regulate iWUE_FL_ response by *OsPIL13*, which supports the fact that stomatal movements could be regulated by some other PIFs in rice^41^ and Arabidopsis^42^.

Previous molecular studies have elucidated the direct regulatory role of PIFs in downstream gene expression by binding to a G-box motif (CACGTG) present in their promoters, including ABI5 and CDF2^26,48^. Based on transcriptome analysis, we identified two extreme DEGs, *OsNHX1* and *OsSAL1*, with downregulated and upregulated levels, respectively, in the *PIL13*^*v3m*^ (Fig. 5A–C). Our study further revealed that *Os*PIL13 directly binds to the G-box motif of both *OsSAL1* and *OsNHX1*, coordinating distinct transcriptional regulation (Fig. 5D, E).

The expression of *SAL1* is stimulated by light, as reported in Arabidopsis^49^, which is in line with our observation that *OsSAL1* expression is inhibited by *Os*PIL13, a light repressor in rice (Figs. 2E, F and 5F). In addition, SAL1 interacts with some light signaling regulators, such as phyB^49^ and HY5, in Arabidopsis^50^. SAL1 is proposed to antagonize the light response of the hypocotyl downstream of a convergence point between phyB and other photoreceptors in Arabidopsis^49^. This reflects the possibility that *Os*PIL13 regulates the expression of *OsSAL1* with downstream regulation by phyB^51^.

Importantly, SAL1-PAP is a typical retrograde signal produced in chloroplasts under DS, triggering the expression of nuclear-encoded stress response genes and facilitating stomatal closure^28,52^. SAL1-PAP pathway also integrates chloroplast retrograde, light, and hormonal signaling in Arabidopsis^53^. Consistent with this, we observed a dramatic degradation in PAP in *OsSAL1-OE* through metabolism analysis. In contrast, the PAP degradation was mitigated in an *OsPIL13*-*OsSAL1* co-overexpression line (Fig. 6D), suggesting that *OsPIL13* participates in the SAL1-PAP mediated chloroplast retrograde signaling pathway and promotes stomatal closure during FL-DS.

Constitutive high-level accumulation of PAP through knocking out SAL1 confers drought tolerance to plants, but slows down and even alters plant growth and development in Arabidopsis^53^ and wheat^54,55^. Notably, compared to less successful DS tolerance through these attempts, we attributed this to a more prominent condition, FL-DS combined stress, than single cases (Supplementary Fig. 12). We hypothesized that under FL-DS, dynamic regulation of *Os*PIL13 on *OsSAL1* expression was essential for maintaining high iWUE_FL_ with optimized carbon gains against stomatal conductances. PAPs, as a signal regulator for stomatal closure^28^, are beneficial for iWUE regulation, especially under FL-DS combined stress, as reported in the current study. Our results support the idea that appropriate stomatal closure might be a key feature to improving plant growth under FL-DS.

In addition, our investigation highlights another gene involved in stomatal closure speed, *OsNHX1*, observed during FL^16^. Importantly, *Os*NHX1 is recognized as a proton-sodium exchanger and has been shown to enhance drought and salt tolerance in Arabidopsis^56^. It could also impact stomatal kinetics during changing environments, such as osmotic stress, daily shifts, and FL, by regulating guard cell turgor and vacuolar K^+^ accumulation in Arabidopsis^57,58^. In our study, *Os*PIL13 could transcriptionally activate *OsNHX1* gene expression (Fig. 5G). The co-overexpression rice line of *OsPIL13* and *OsNHX1* markedly favored plant growth under DS by hastening stomatal response speed during FL (Fig. 6F–I; Supplementary Fig. 16C, D). This observation suggests that the *OsPIL13*-*OsNHX1* module contributes to the accumulation of K^+^ ions within plant cell vacuoles, thereby augmenting their osmotic potential. This process drives the water uptake, generating the requisite turgor pressure essential for cell expansion and growth under DS^58^.

Plants always exhibit enormous diversity in some water-conserving traits during FL^14,59^; these plant water-conserving traits are well adapted to their local light circumstances^16,60^. We compared the distribution of *OsPIL13* haplotypes with local light regimes from where rice accessions were originally collected, where most *japonica* rice accessions preferentially exist in Hap II, representing the elite haplotype with high iWUE_FL_. These rice accessions were majorly derived from the regions characterized by low solar irradiance and annual visibility (Figs. 3D and Supplementary Fig. 20A–E), suggesting that the elite haplotype of the *OsPIL13* gene in the *japonica* rice group might have undergone artificial selection targeted for high iWUE_FL_ through stomatal closure under low light regime, rather than *indica* rice group. However, the negative effect of rapid stomatal closure, which increases the risk of stomatal closure limitation, is lethal and non-negligible during FL^61^. Notably, the selection pressure might be higher in northern areas of America, Japan, and China (Fig. 7F). Hence, the rapid stomatal responsiveness during FL might result from indirect breeding selection for better growth and yield potential under DS, similar to other species, such as sugar beet^62^.

## Conclusion

Our results demonstrate that iWUE_FL_ is closely related to FL-DS tolerance, with high SNP heritability. Based on GWAS, we identified the *OsPIL13* gene strongly associated with the variation in iWUE_FL_ in both field and GC conditions. Overexpressing either *OsPIL13* or its homolog in soybean, *GmPIL13*, led to at least a 10% increase in iWUE_FL_. We confirmed a v3 SNP at the *OsPIL13* promoter region as a candidate causal allele for regulating iWUE_FL_. *Os*PIL13 transcriptionally represses *OsSAL1* and activates *OsNHX1* expression by directly binding to the G-box motifs at promoters of these two genes, and hence promotes iWUE_FL_ during FL-DS. We found that *japonica* rice accessions preferentially harbor the elite haplotype group of *OsPIL13* (Hap II), which is always distributed in geographic regions with low light regimes. These findings provide new insights into the molecular mechanism by which *Os*PIL13 regulates iWUE_FL_, collaborating with the SAL1-PAP signaling and the NHX1 ion exchanger. Our study underscores the importance of engineering the *OsPIL13* causal allele for molecular breeding aimed at improving yield under FL-DS in rice, which also offers valuable guidance for the molecular breeding of FL-DS tolerance in other species, including soybean.

## Materials and methods

### Materials

The Minicore rice population, comprising 206 accessions from 97 countries, was utilized in this study. The population was categorized into six subpopulations: *indica* (IND, 35.4%), *aus* (AUS, 18.7%), *tropical japonica* (TRJ, 18.2%), *temperate japonica* (TEJ, 15.2%), *aromatic* (ARO, 3.0%), and admixtures (Admix, 9.6%)^15,63^. In a superclass, *japonica* includes TEJ, TRJ, and ARO, while *indica* includes IND and AUS. The population represents a significant genetic variation and an appropriate population size, making it an ideal germplasm for identifying photosynthetic traits^16^.

### Fluctuating light and drought stress combined treatments

To investigate the natural variation of iWUE kinetics induced by FL in combination with DS (FL-DS) in a rice population, two independent experiments were conducted under both field and growth chamber (GC) conditions. For the field experiment, the population was cultivated in a paddy field in Yazhou, Hainan, China (110.0375°E, 18.5060°N) on November 18^th^, 2020, using a randomized complete block design with four replications. The soil composition in field plots was maintained at around 20% sandy soil containing small particles of weathered rock, and 80% paddy soil, thus minimizing the effects of plant movement on root damage for iWUE measurements^64^. To minimize boundary effects, a planting scheme of 49 plants for each rice accession was implemented (7 × 7), with 15 cm spacing between plants within each row and 20 cm between rows. Standard local agronomic practices were followed for field management. To investigate the iWUE kinetics effects of FL-DS in the Minicore population, plants were carefully transferred to pots in a growth chamber (GC) at around 40 days after germination (DAG), then exposed to either FL-DS or well-watered conditions under HL as a control (CK) for 20d. Four rice seedlings of each accession were grown per pot, and two pots were used per accession, which signifies that at least four replicates were used for statistical analysis. DS treatments were applied by irrigating every 5d. Soil humidity was maintained at 25–40% and monitored using a soil moisture meter (Hansatech Instruments Ltd, UK). A FL treatment cycle of 40 min comprises of 10 min of HL (1500 μmol m^−2^s^−1^), 25 min of low light (LL, 100 μmol m^−2^s^−1^), and 5 min of HL as reported previously^6^. The frequency of FL was controlled by a light frequency controller (FL053C, Zhongkang Omics Corp. Ltd., China). The FL treatments were conducted each day from 6:30 am to 6:30 pm. The CK was performed to estimate the biomass ratio values of rice accessions in the Minicore population to determine the tolerance to FL-DS.

For GC experiments, rice seeds were sown for ∼10 d in a soil seed bed, then transplanted into 12 L plastic pots containing commercial peat soil (Pindstrup Substrate no. 4, Pindstrup Horticulture Ltd, Shanghai, China) and kept in outdoor conditions to ensure normal growth. Four rice seedlings of each accession were grown in each pot, with two pots used per accession. FL-DS combined treatment was applied from 40 to 60 DAG with 33/27 °C day/night temperature, a 12 h photoperiod, and 60-70% air humidity. The same FL-DS treatment protocol was followed, and the field transfer to GC approach was followed to ensure the consistency between field and GC conditions.

### Kinetics of iWUE measurements during FL

To ensure stomatal adaptation, a 2 h HL treatment (1500 μmol m^−2^s^−1^ photosynthetic photon flux density, PPFD) was applied before measuring iWUE kinetics^6^. For iWUE kinetics measurements, four replicates were conducted simultaneously using a high-efficiency all-weather photosynthetic measurement system (HAPS)^33^, coupled with four portable photosynthesis systems, LICOR 6400 (Li-COR, Inc.). To minimize potential errors introduced by growth stage differences, photosynthetic parameters were measured sequentially from accession 1 to 206 for the first and third replicates, and from accession 206 to 1 for the second and fourth replicates^6^. Measurements were taken during FL treatment between 9:30 to 16:30. The FL measurement in the leaf cuvette followed the same light transition cycle as the GC setup, controlled by an automatic program in LICOR 6400. Several gas exchange parameters measured under high HL (1500 μmol m^−2^s^−1^ PPFD) include net *A*, *g*_s_, and iWUE. Fully expanded leaves were used, with CO_2_ concentration maintained at 400 μmol mol^−1^ and flow rate at 500 μmol mol s^−1^. Leaf cuvette humidity was maintained at ~70%.

### Calculation of iWUE parameters

The iWUE dynamics during light transitions occur in distinct phases^6^. As the light shifts from high to low intensity (from time-point *i* to *j*), iWUE decreases sharply. From time-point *j* to *k*, the iWUE linearly increases due to the *g*_s_ reduction. From time-point *k* to *p*, a slight iWUE drop occurs due to *g*_s_ recovery. Upon HL resumption (from time-point *p* to *f*), iWUE increases rapidly. To quantify these transitions, five iWUE-related parameters are calculated using specific equations:

1 $${iWUE}=\frac{A}{{{\rm{gs}}}}$$

2 $${{iWUE}}_{{Ir}}={\sum }_{n=j}^{n=k}\left(\frac{\Delta T\times \Delta {iWUE}}{k-j}\right)$$

3 $${{iWUE}}_{{Dr}}={\sum }_{n=K}^{n=P}\left(\frac{\Delta T\times \Delta {iWUE}}{p-k}\right)$$

4 $$i{{WUE}}_{{LL}}=\frac{\sum {iWUE}5}{5}$$

5 $$i{{WUE}}_{{FL}}=\frac{\sum {iWUE\; during\; FL}}{\Delta T}$$

Where Δ*T* represents the time interval from each point during FL, iWUE_Ir_ signifies the slope of iWUE during the linear increase phase of FL (from *j* to *k*). Meanwhile, iWUE_Dr_ represents the slope of iWUE during the third stage of decreasing light (from *k* to *p*). iWUE_LL_ denotes the steady-state iWUE calculated from five peaked values during the low-light phase^6^. iWUE_FL_ refers to the averaged iWUE during a FL cycle. We defined iWUE_FL_ as a balance index between photosynthesis and water loss via stomata. MATLAB v.R2010a (Mathworks Inc., Natick, MA, USA) was used to fit stomatal closure kinetics to estimate τ_cl_ with a first-order exponential decay curve^65^.

### Genome-wide association study

A genome-wide association study (GWAS) was performed using the genotype dataset obtained from low-coverage genome sequencing^27^. GEMMA software v.0.98.5, employing a mixed linear model, was used for the analysis^66^. A relatedness matrix and the first four principal components derived from the principal component analysis (PCA) were incorporated to account for population structure. To prevent inflation in the genome-wide association tests, a genome-wide significance level was set using 200 permutations of phenotypes to determine cut-off^27^. Manhattan and QQ plots for GWAS were generated using the R package qqman^67^. LocusZoom plots were generated using an R script sourced from GitHub (https://github.com/statgen/ locuszoom-standalone).

### Haplotype analysis

Linkage disequilibrium (LD) analysis was conducted using Haploview v.4.2 to explore the genetic relatedness of candidate genes near the lead SNP. LD blocks were defined by criteria: the upper 95% confidence bounds of the *r*^2^ value surpassing 0.98 and lower bounds exceeding 0.70^68^. Genes within these LD blocks were considered as potential candidate genes associated with iWUE-related traits.

### Estimation of trait heritability

Trait heritability (*h*^2^_SNP_) indicates the proportion of phenotypic variance explained by SNPs^69^. The *h*^2^_SNP_ for iWUE-related parameters was estimated using GCTA v.1.11.2 beta via the Restricted Maximum Likelihood method. The *P*-value of SNP heritability was assessed using the Log Likelihood Test (LRT) per the GCTA software manual.

### GO and KEGG analysis

An online tool AgriGO v2 (https://systemsbiology.cau.edu.cn/agriGOv2/)^70^ was utilized for gene ontologies (GO) annotation of candidate genes corresponding to SNPs overlapping among various iWUE parameters, referencing the UniProtKB GOA file (ftp.ebi.ac.uk/pub/databases/GO/goa). The KOBAS software (KEGG Orthology Based Annotation System, v2.0) was utilized to identify reprogrammed biochemical pathways^71^.

### Generation of transgenic plants

To investigate the function of *OsPIL13* (*LOC_Os03g56950*) based on GWAS, two mutants of *OsPIL13* were generated: 1) to validate the biological function of v3 SNP, a locus specific mutant line of *OsPIL13* (named *PIL13*^*v3m*^) was generated with a one-nucleotide knockout targeting a v3 SNP. 2) a knock-out mutant of *OsPIL13* (*ospil13*) at its third exon through two nucleotides “AA” insertion, leading to an early stop codon formation at 270^th^ amino acid. Additionally, a mutant of its regulatory gene *OsSAL1* (inositol polyphosphate 1-phosphatase, *LOC_Os07g37220*) was generated with two nucleotides (“CT”) knockout at 17-bp after the start codon, leading to early translation termination at 55^th^ amino acids, named *ossal1*. These mutant creations were accomplished using CRISPR/Cas9 technology implemented by the Biogle Company (Hangzhou, China). The single guide RNA (sgRNA) was inserted into a BGK032-DSG vector containing Cas9. This vector was introduced into an *Agrobacterium tumefaciens* (thereafter, *A. tumefaciens*) strain EHA105 and then transformed into wild-type Wuyungeng 7 (WYG7, termed WT), a modern rice cultivar belonging to Hap II. A mutant of *OsNHX1* (*osnhx1*) was also used in the current study^16^. Homozygous lines from this process were sequenced based on gene-specific primers (Supplementary Table 1).

For the construction of the overexpression vector for *OsPIL13, OsSAL1*, and *OsNHX1* (vacuolar sodium/proton antiporter, *LOC_Os07g47100*), their cDNA (primer sequences listed in Supplementary Table 1) was amplified from WYG7. Restriction sites (*Bam*HI and *Sac*I) were added, and the amplified fragments were transferred into the pCAMBIA 1301 plasmid backbone. This plasmid backbone included a GFP tag (Youbio, China, VT1842) and the hygromycin B phosphotransferase (*HPT*) gene driven by its native promoter from HapII (*pOsPIL13*^*HapII*^*::OsPIL13-GFP*) to generate *OsPIL13*-OE rice lines. The three vectors were then individually transformed into rice^72^ to generate overexpression lines of different genes (OE). Additionally, their native promoters were used to construct *OsSAL1* and *OsNHX1* overexpression lines. Co-overexpression lines of two genes, including *OsPIL13*-*OsSAL1* and *OsPIL13*-*OsNHX1*, were obtained by crossing the OE lines of *OsPIL13* with other OE lines of either *OsNHX1* or *OsSAL1*.

Positive OE lines of three genes were detected using primers listed in Supplementary Table 1. Three homozygous OE lines with the highest expression of the *OsPIL13* gene and one homozygous OE line for *OsNHX1* and *OsSAL1* from the T_3_ generation were selected for FL-DS treatment experiments. Primers for detecting the *HPT* expression level are provided in Supplementary Table 1.

Soybean typically experiences the FL-DS condition during maize-soybean intercropping. To investigate the biological function of *OsPIL13* homolog (*GmPIL13*, *Glyma.08G303900*) in soybean, we also generated a mutant (knock-out) line and two overexpression lines of *GmPIL13*. The knockout construct of *GmPIL13* was designed using CRISPR-Cas9 technology^73^. Knockout mutant was generated with a 20-bp target sequence at the first exon of *GmPIL13* for a Cas9 cleavage site. Primers used for detecting mutation sites are illustrated in Supplementary Table 1. The CRISPR-Cas9 plasmid was transformed into the soybean cultivar Dongnong 50 (DN50), and the generated transgenic plants were selected using the Bar resistance marker. For the overexpression method, the ORF (open reading frame) of *GmPIL13* was obtained from DN50, amplified by overlapping PCR to obtain one fragment, and then introduced into the pTF101-Gene vector (containing the bar gene for glufosinate resistance)^74^. The construct driven by its native promoter (*pGmPIL13*:*GmPIL13-GFP*) was then transformed into *A. tumefaciens* strain EHA105, and thus the transgenic lines were generated by *A. tumefaciens*-mediated transformation using the floral dip method with DN50 accomplished by Weimi Biotech. Company (Hainan, China), and then 1/500 10% (w/v) Basta (Ingbio, Lot: CB26213210) selection.

### Stomatal aperture determinations

The stomatal aperture determination was assessed on 20d after FL-DS, where leaves were sampled during different time-points (0, 10, 20, 30, and 60 min) during FL. The top fully expanded leaves from each plant were collected and stored in formalin-acetic-alcohol (FAA) fixative solution for subsequent analysis. Stomatal observation protocols were employed^25^. Using a TM-1000 desktop scanning electron microscope, stomatal characteristics, including stomatal width (W_stomata_) and stomatal length (L_stomata_) representing the characteristic dimensions (width and length), of stomatal pores on the adaxial leaf surface, were observed and recorded.

### Determination of *Os*PIL13 subcellular localization

To assess *Os*PIL13 subcellular localization, we implemented a transient transformation in tobacco leaves. A GFP fusion vector, *pOsPIL13*^*HapII*^*::OsPIL13-GFP*, was constructed using the pCAMBIA1300 backbone. The known nuclear-localized bZIP transcription factor EmBP1 was used as a positive control to validate nuclear localization^35^. The transformation of the construct into tobacco leaves was achieved through the agro-infiltration method^75^. Briefly, the GFP-protein fusion construct was transfected into *A. tumefaciens* strain C58C1 (WeiDi Biotech, China, AC1110). Subsequently, it was transiently expressed in the leaf epidermal cells of 5-week-old tobacco (*Nicotiana benthamiana*) plants through *Agrobacterium*-mediated leaf infiltration^76^. A visual observation was performed using confocal laser scanning microscopy (Zeiss LSM 700, Germany) equipped with a Fluor 10X/0.50M27 objective lens and an SP640 filter. The green fluorescence of the GFP fusion protein was excited at 488 nm, and signals were collected at 660–736 nm for chlorophyll autofluorescence and 495–515 nm for GFP.

### RNA extraction and qRT-PCR analysis

The top fully expanded leaves from each plant at 40 DAG, either unexposed (CK) or exposed to 20d FL-DS, were collected for qRT-PCR analysis. Total mRNA extraction was performed using TRIzol Reagent (Invitrogen), followed by the removal of genomic DNA using DNase I treatment (Takara). The extracted RNA was reverse transcribed into cDNA using the SuperScript VILO cDNA Synthesis Kit (Invitrogen Life Technologies). We employed SYBR Green PCR Master Mix (Applied Biosystems, USA, 4309155) for qRT-PCR analysis on a Real-Time PCR System (ABI StepOnePlus, Applied Biosystems, USA). Primers for qRT-PCR were designed using Primer Prime Plus 5 Software v. 3.0 (Applied Biosystems, USA), and the *Actin1* gene (*LOC_Os03g50885*) served as an internal reference. The relative expression of the gene against *Actin1* was quantified using the 2^−ΔΔCT^ method (ΔΔCT = CT, gene of interest^−CT^)^77^. This analysis was performed based on three biological replicates. The primers were employed to determine gene expression levels responsive to iWUE_FL_ (Supplementary Table 1).

### Transcriptome analysis

For transcriptome analysis of WYG7 and *PIL13*^*v3m*^ leaves, RNA degradation and contamination were monitored using 1% agarose gel electrophoresis, and purity was assessed using a Nano-Photometer spectrophotometer (IMPLEN, CA, USA). The RNA Nano 6000 Assay Kit on the Agilent Bioanalyzer 2100 system (Agilent Technologies, CA, USA) was employed to evaluate RNA integrity. Subsequently, 1.5 μg of RNA per sample was used for RNA sample preparations. The NEBNext Ultra RNA Library Prep Kit for Illumina (NEB, USA) was utilized to generate sequencing libraries^78^. Following cluster formation, the prepared libraries were sequenced on an Illumina HiSeq 4000 platform, producing 150 bp paired-end reads.

The quality assessment of RNA-seq data was performed using FastQC software. Following generation of genome index, clean RNA-seq reads were aligned using STAR^79^, with the ‘—quantMode GeneCounts’ option utilized to quantify the number of reads per gene. Subsequently, gene and isoform quantification were carried out using Cufflinks v.2.2.1. Therefore, differentially expressed genes (DEGs) analysis between WYG7 and *PIL13*^*v3m*^ under FL-DS conditions was performed using the fragment per kilobase of transcript per million mapped reads (FPKM) method to assess transcript abundance. Then, DEGs were identified employing the R package ‘DESeq2’^80^, with read counts obtained from STAR^79^ considered in the analysis. Only genes exhibiting an adjusted *P*-value < 0.05 were considered as DEGs. To minimize transcriptional noise, each isoform/gene was included for analysis if its FPKM values were >0.01, based on a threshold established through gene coverage saturation analysis^66^.

### ChIP-qPCR assays

All leaves of rice seedlings (*OsPIL13* OE1 transgenic plants and WYG7) grown under FL-DS conditions for 20d were used for the chromatin immunoprecipitation (ChIP) assays^81^. Approximately 2.5 g of leaf blades from three seedlings were harvested for each replicate to extract chromatin. In this regard, three biological replicates were conducted. After sonication, the supernatant was incubated with GFP-Trap magnetic agarose beads (Chromotek, Martinsried, Germany) at 4 °C for 1.5 h. An eGFP coding sequence was amplified from the 35S-eGFP construct to generate eGFP-pBI101.3 as a negative control. Enrichment efficiency was assessed as a percentage of IP DNA relative to input DNA. Primers used for ChIP-qPCR are also given in Supplementary Table 1.

### Luciferase activity assay in rice protoplasts

To analyze the response of the *OsPIL13* promoter to FL, ~2.0-kb DNA fragments upstream of the *OsPIL13* coding region were amplified from T4178 and A4003 and inserted into the pGreenII 0800-LUC vector, generating pHapI-LUC and pHapII-LUC, respectively. Subsequently, six variants were generated by mutating pHapII-LUC using the Q5 site-directed mutagenesis kit (NEB, E0552S). The primer sets used for PCR amplification and mutation are detailed in Supplementary Table 1. One-month-old rice seedlings of WYG7 grown under a high light regime (~1500 μmol m^−2^s^−1^ PPFD) underwent a 24 h dark exposure before leaf sample collection for protoplast extraction. The generated vectors were individually transformed into protoplasts, then divided into two groups and exposed to either dark or high light conditions for 1 h to establish distinct light environments. All samples were incubated in cell lysis buffer (10 mM Tris-HCl, pH 8, 2 mM MgCl_2_) for 4–6 h at 28 °C. The activities of firefly luciferase (LUC) and *Renilla* luciferase (REN) were assessed using the Dual-Luciferase Reporter Assay System kit (Promega, E1960). The ratio of LUC/REN was calculated as relative activity, and the dark/HL ratio was used to characterize light response. Each vector underwent three replicates to evaluate its light responsiveness.

### Yeast-one hybrid assays

Yeast one-hybrid (Y1H) assays were conducted to investigate *Os*PIL13 binding to the G-box motif (CACGTG) within *OsNHX1* and *OsSAL1* promoters (Clontech, CA, USA). The coding region of *OsPIL13* was amplified and cloned into the GAL4 activation domain (GAL4 AD) of pGADT7-Rec2 (Clontech, CA, USA), forming the prey vector. Simultaneously, ~1.5-kbp segments upstream of the ATG start codon for *OsSAL1* and *OsNHX1*, containing the G-box CACGTG-motifs, were amplified and cloned into the pAbAi vector (Clontech, CA, USA) to generate the Pro-*OsSAL1*-AbAi and Pro-*OsNHX1*-AbAi bait vectors. Additionally, mutated motifs were generated via PCR and ligated into pAbAi. These prey and bait vectors were co-transformed into the Y1H Gold yeast strain. The positively transformed yeast cell concentrations, harboring various bait-prey vector combinations, were adjusted to OD_600_ ≈ 1.0 and then serially diluted 1/10, 1/100, and 1/1000 in sterile ddH_2_O. The serial dilution of transformed yeast cells was cultured on SD/−Leu medium plates with an optimal concentration of aureobasidin A (AbA) to examine protein-DNA interactions. An empty vector containing recombinant GFP was co-transformed as a negative control.

### PAP content determinations

Total adenosines were extracted using 0.1 M HCl, followed by derivatization with chloroacetaldehyde. Quantification was conducted fluorometrically after HPLC fractionation^82^. Quantifying 3′-phosphoadenosine 5′-phosphate (PAP) involved integrating the HPLC peak area and converting these to pmol units using standard curves calibrated with 1, 5, and 10 pmol standards.

### Western blot analysis

Western blot experiments were conducted to compare the protein levels of *Os*PIL13 and *Os*SAL1 in their OE lines. Approximately 5 µg of total protein from crude leaf extracts was used, and the protein concentration was determined using the Bicinchoninic Acid (BCA) protein concentration assay kit (Beyotime, P0010). The extracts were loaded and separated on a 12% SDS–PAGE gel. Subsequently, the gel was either stained with Coomassie Brilliant Blue (CBB) or transferred to a nitrocellulose membrane for Western blot analysis^83^. Signals were detected using a Pierce ECL Plus Kit (Thermo Scientific, USA) and visualized with a luminescent image analyzer (Tanon-5200, Tanon). Antibodies against *OsPIL13* (cat#AS163955), *OsNHX1* (cat#AS09484), *OsSAL1* (cat#AS07256) from Agrisera (USA) and anti-GFP (A-11122, Invitrogen) were used at a dilution of 1:10,000.

### Statistics and reproducibility

Data from all biological triplicate experiments are presented with error bars as mean ± SD. Two-tailed unpaired Student’s *t* test was used to compare the two cohorts of data. One-way *ANOVA* or two-way *ANOVA* was performed to compare multiple cohorts of data. *P*-value of less than 0.05 was considered statistically significant. All statistical analyses were performed using GraphPad Prism 10.

## Supplementary information

Supplementary Information

Description of Additional Supplementary Files

Supplementary Data 1

Supplementary Data 2

Supplementary Data 3

Reporting-Summary

## Data Availability

The GEO number of RNA sequencing raw data is GSE284707. The detailed processed data of RNA sequencing were deposited in Supplementary Data 2. Supplementary Fig. 22 lists all Western blot experiments in the manuscript in uncropped and unedited forms. All source data are included in the “Supplementary Data 1, 2” file linked to this manuscript. The data used to substantiate the findings of this study are retrievable from the author at a reasonable request.
