# Supplementary material for: The phytochrome-interacting factor PIL13 enhances water use efficiency under fluctuating light and drought resilience in rice and soybean
Source: Commun Biol. 2025 Aug 26;8:1286. doi: 10.1038/s42003-025-08605-8 (PMC12381223; doi:10.1038/s42003-025-08605-8)
Supplement: Supplementary file 1 — Supplementary Information [file 42003_2025_8605_MOESM1_ESM.pdf]

Supplementary Note 1. Investigation of five iWUE dynamic traits in the Minicore population and validation of $iWUE_{FL}$ as a key indicator for FL-DS response

Identifying photosynthetic traits with high heritability would be helpful for molecular assisted-selection for precise breeding targeted with high photosynthetic efficiency under various abiotic stresses. iWUE presents an essential parameter for drought breeding. Still, experience has shown that improvements in iWUE may not be always translateed into higher crop water-use efficiency (WUE) or yield due to external environmental changes[1]. This study considered the effects of changing light regimes on iWUE. We performed large-scale iWUE dynamics measurements under FL-DS condition using a rice Minicore population in both field and growth chamber (GC) conditions. The structure and genomic sequence information of the Minicore population were previously published [2]. To ensure normal growth, rice seeds were firstly sown in a paddy field, using a randomized complete block design, and then transferred to pots in a GC at around 40 days after germination (DAG), with an exposure to either FL-DS or CK for 20 days as detailed in material and method section. As earlier reported, a DS treatment was conducted through interval irrigation by watering every 5 d according to the soil humidity records[3, 4]. FL treatment of 40 min for each cycle consists of 10 min of high light (HL, 1,500 μmol $m^{-2}s^{-1}$), 25 min of low light (LL, 100 μmol $m^{-2}s^{-1}$), 5 min of HL[5]. To record the iWUE dynamics, an auto-programmed FL in a leaf cuvette was controlled with 10 min HL, 25 min LL, and 5 min HL. As documented previously, the 25 min LL was used to obtain the steady-state stomatal conductance through the rice Minicore population[3, 4]. Histograms of normalized phenotypic values were evaluated for the five iWUE traits (Supplementary Fig. 1).

To ensure the experimental reproducibility, we performed a GC condition to mimic the field conditions by manually controlling the FL. As reported above, the FL-DS combined treatment was also performed. Temperature (day/night) was maintained 33/27°C, while air humidity was controlled ranging of 60~70%.

In our experimental design, each of the 206 rice accessions was applied to two pots, with two plants per pot measured independently, yielding four observations per rice accession. To exclude the pots effects of iWUE measurements for both field and growth chamber conditions, we performed detailed variance analysis, and results confirmed that between-pot variation

within rice accessions was statistically non-significant (field: F = 1.1, p = 0.2167; and for growth chamber: F = 0.96, p = 0.6352). This indicates that the observed differences primarily reflect treatment effects rather than pot-level variability.

Analysis of Variance Table for $iWUE_{FL}$-GC

Source of Variation	Degrees of Freedom (df)	Sum of Squares (SS)	Mean Square (MS)	F-ratio	p
Between Accessions	193.00	28488.53	147.6089637	6.551213182	1.53E-26
Between Subgroups (Within Accessions)	194.00	4371.12	22.53154639	0.956120502	0.6352
Within Subgroups (Pot error)	388.00	9143.45	23.56559278	-	
Total	775.00	42003.1		-	

Analysis of Variance Table for $iWUE_{FL}$-field

Source of Variation	Degrees of Freedom (df)	Sum of Squares (SS)	Mean Square (MS)	F-ratio	p
Between Accessions	193.00	36467.38	188.9501554	7.497408606	4.27E-39
Between Subgroups (Within Accessions)	194.00	4889.2	25.20206186	1.100785648	0.216662
Within Subgroups (Pot error)	388.00	8883.11	22.8946134	-	
Total	775.00	50239.69		-	

Traits with significant phenotypic variation, high heritability, and low environmental plasticity are essential features for breeding target selection. All five iWUE traits exhibited huge natural variations in the Minicore population and each subpopulation (Supplementary Figs. 1-2). In addition, we observed a strong positive correlation between $iWUE_{FL}$ in the field and that ($iWUE_{FL}$) in GC, with Pearson correlation coefficient (R) >0.7 (Supplementary Fig. 3F). Consistently, plants with different $iWUE_{FL}$ values maintained a similar ranking between field and GC conditions (Fig. 1B). Furthermore, we found that both $iWUE_{FL}$ (field and GC) have highest SNP heritabilities in the five iWUE traits for both field and GC conditions (Fig. 1E). Collectively, we deduce that $iWUE_{FL}$ could be an essential photosynthetic trait with a high

heritability used for molecular selected-breeding.

Supplementary Note 2. GWAS on five iWUE traits using low-coverage genotype dataset

To assess the potential of our GWAS design for causal gene identification, we performed a genome-wide association study (GWAS) for the five iWUE dynamic traits. Determining substantial SNP peak utilized a threshold established through 200 permutations[2]. Given the strong correlations observed between iWUE-related parameters, an analysis of overlapping SNPs associated with these traits was conducted (Fig. 1F-G; Supplementary Fig. 4A-D).

In general, 665 SNPs in total were found above the threshold across the five iWUE traits (Supplementary Fig. 4E). These SNPs were positioned at Chromosomes 1, 2, 3, 5, and 12 (Supplementary Fig. 4E). Within the overlapped genomic window, we identified 66 candidate genes within the surrounding 50-kb regions of these 20 overlapping SNPs. Correspondingly, we found some reported QTLs-based genes related to photosynthetic traits were co-identified in the five iWUE dynamic traits, including *PsbP*[6], *PIL13*[7], *Psb28*[8], and $qD_{ada}12$[9] (Supplementary Fig. 4A-D). Subsequent gene ontology (GO) analysis revealed significant enrichment in biological processes such as xylan catabolic process, proteolysis, and protein catabolic process, as well as molecular functions including aspartic-type endopeptidase activity and hydrolase activity acting on glycosyl bonds among the identified 66 candidate genes (Supplementary Fig. 4G).

Importantly, we identified six genes among the 66 candidate genes that were co-identified to be strongly associated with both $iWUE_{FL}$ under both field and GC conditions (Fig. 2A-B). The candidate genes share genomic regions within the same linkage disequilibriumm(LD) block, although the lead SNPs differ between the two conditions (lead SNP is 3m32427037 for field and lead SNP is 3m32401540 for GC). The two lead SNPs explained about 20% of the phenotypic variation (Fig. 1F-G). Given their association with FL response regarding $iWUE_{FL}$, we examined the transcript expression of the six candidate genes. We found that only *OsPIL13* (LOC_Os03g56950) showed differences in expression levels between the rice subgroups consisting of 10 accessions with high $iWUE_{FL}$ and 10 with low $iWUE_{FL}$ (Fig. 2D). Furthermore,

we identified 15 SNPs at genomic regions of *OsPIL13* with a low-coverage genotype dataset (Supplementary Table 3).

Supplementary Note 3. Validation of the association signal using a high-coverage genotype dataset

To fully resolve the DNA sequence variation in *OsPIL13* that might have been missed by low-coverage genome sequencing[2], we re-sequenced *OsPIL13.* We identified 46 SNPs and 6 insertions and/or deletions (indels) (Supplementary Data 1). After conducting an association analysis with the identified variants, we confirmed nine SNPs with P value $<1.86\times10^{-6}$ (Fig. 3A; Supplementary Table 4). The nine SNP variants were included in the low-coverage genotype dataset, suggesting the importance of these variants used to perform further causal variant analysis.

List of Supplementary Figures and Tables

Supplementary Table 1. List of primers used in this study.

Supplementary Table 2. Detailed information of rice accessions with contrasting $iWUE_{FL}$ in Minicore rice population.

Supplementary Table 3. Frequency of major allele frequencies of SNPs surrounding *OsPIL13* based on low-coverage sequencing dataset in Minicore rice population.

Supplementary Table 4. Haplotype analysis on allelic variation of nine SNPs at *OsPIL13* gene possessing significant association with $iWUE_{FL}$.

Supplementary Table 5. Statistical analysis of the reads based on transcriptome analysis.

Supplementary Figure 1. Distribution of five iWUE parameters in the rice Minicore population exposed to FL-DS combined treatment in field.

Supplementary Figure 2. Distribution of five iWUE parameters in the different subpopulations of the Minicore panel exposed to FL-DS treatment in field.

Supplementary Figure 3. Pearson correlation analysis among the five iWUE parameters and biomass ratio in FL-DS against CK condition.

Supplementary Figure 4. Overlapped SNPs identified in different iWUE traits based on GWAS.

Supplementary Figure 5. Spatiotemporal analysis of *OsPIL13*.

Supplementary Figure 6. Relative gene expression levels of *OsPIL13* in response to ABA and circadian rhythm patterns.

Supplementary Figure 7. The zoom-in Manhattan plot of the nine SNPs at the *OsPIL13* gene associated with $iWUE_{FL}$ under both GC and field conditions.

Supplementary Figure 8. Knocking-out *OsPIL13* leads to reduced $iWUE_{FL}$ under FL-DS.

Supplementary Figure 9. Dynamics of A and g_s in WYG7 and $PIL13^{v3m}$ exposed to 20 d FL-DS treatment.

Supplementary Figure 10. Physiological and agronomic traits of *OsPIL13* overexpression lines under FL-DS condition.

Supplementary Figure 11. Performance of *OsPIL13* transgenic rice lines under CK conditions.

Supplementary Figure 12. Greater enhancement of $iWUE_{FL}$ by *OsPIL13* under FL than under DS.

Supplementary Figure 13. *Os*PIL13 specifically binds to the G-box motif present in the *OsSAL1* promoter.

Supplementary Figure 14. *Os*PIL13 specifically binds to the G-box motif of the *OsNHX1* promoter.

Supplementary Figure 15. Dynamics of A and g_s during FL-DS condition for WYG7 and *ossal1*, *OsSAL1*-OE, and a co-overexpression line of *OsPIL13* and *OsSAL1*.

Supplementary Figure 16. Dynamics of A and g_s during FL-DS condition between WYG7, *OsNHX1*-OE, and a co-overexpression line of *OsPIL13* and *OsNHX1* under FL-DS condition.

Supplementary Figure 17. Gene expression in mutants of *OsSAL1* and *OsNHX1* exposed to FL-DS.

Supplementary Figure 18. Effects of *Os*PIL13 on *OsSAL1* in regulating iWUE under CK condition at the graining stage.

Supplementary Figure 19. Growth performance of WYG7, *OsNHX1*-OE, and a co-overexpression line of *OsPIL13* and *OsNHX1* under CK condition.

Supplementary Figure 20. Geographical distribution and original location climate of *OsPIL13* haplotypes in the Minicore population.

Supplementary Figure 21. *Gm*PIL13 promotes $iWUE_{FL}$ and biomass accumulation under CK condition in *Glycine max*.

Supplementary Figure 22. Raw, uncropped data of gels for each data.

Supplementary Tables

Supplementary Table 1. List of primers used in this study.

Gene abbrev.	Primer sequences (5'-3')	Purpose
APP1-F	CATTTGTGCCGCCGATTGAT	qPCR
APP1-R	CTATGGCACGGGTGGAACG	qPCR
HP1-F	CCTACAAAAAGGGCGGCAAG	qPCR
HP1-R	AACAAAGGGGGCATGCCATT	qPCR
LRAP19-F	CCGAAGGTGTCGTTGCCA	qPCR
LRAP19-R	CACGTTCACGGTCGGGATG	qPCR
PPR-F	TGATGGTTTGTGTCGGGAGG	qPCR
PPR-R	TGCTTCCCTAAACGCCTCTG	qPCR
Actin-F	AAGCTCATGCCGACCAGAAA	qPCR
Actin-R	AGCCCAAAATGTGGGGTTGA	qPCR
OsPIL13-F	TGAGAGAGGCAAGCAATCCC	qPCR
OsPIL13-R	GCTAGCTGACGCTTTCGGTA	qPCR
NHX1-F	GTAATACGACTCACTATAGGGC	qPCR
NHX1-R	ACTATAGGGCACGCGTGGT	qPCR
SAL1-F	CACCTCGCACAGCGTCCTCA	qPCR
SAL1-R	GCAATCTCTTGGCATCTTGCCTC	qPCR
Actin1-F	GGACCAGCAGAGGTTGATCT	qPCR
Actin1-R	AATGGTGTCCGAGCTCTCAA	qPCR
Tublin-F	TCTTGGACAACGAAGCCATCT	qPCR for soybean
Tublin-R	GGTGAGGGACGAAATGATCT	qPCR for soybean
OsPIL13-v3m-F	GCTGTGATGAAAGCTCTCGC	CRISPR/CAS9 detection
OsPIL13-v3m-R	CGCCGAACGATGAATGCAG	CRISPR/CAS9 detection
GmPIL13 mutant-F	TGAACAACAGTGTTCCTGATTGGAA	CRISPR/CAS9 detection
GmPIL13 mutant-R	AGCTCCACAAGCTCTTGGTC	CRISPR/CAS9 detection
Hygromacin-F	AGCTGCGCCGATGGTTTCTACAA	Overexpression line detection
Hygromacin-R	ATCGCCTCGCTCCAGAGTCAATG	Overexpression line detection
Pro-NHX1-AbAi-F	AAATGATGAATTGAAAAGCTTTGAGTGAAATTTG ATGTGTT**TGACG**	Y1H
Pro-NHX1-AbAi-R	AGCACATGCCTCGAGGTCGACCAAATAGTTTGAG AAGTGTACAA**CGTCA**	Y1H
Pro-NHX1-m-AbAi-F	AAATGATGAATTGAAAAGCTTTGAGTGAAATTTG ATGTGTT**TctCt**	Y1H

Pro-NHX1-m-AbAi-R	AGCACATGCCTCGAGGTCGACCAAATAGTTTGAG AAGTGTACAA**aGagA**	Y1H
Pro-SAL1-AbAi-F	GCTACTCTACTGGTCGTCCGTCAT	Y1H
Pro-SAL1-AbAi-R	GCTGCCAGGGGCGAAACTCTGA	Y1H
Pro-SAL1-m-AbAi-F	GCTACTCTACTGGTCGTCCGTCAT	Y1H
Pro-SAL1--m-AbAi-R	GCTGCCAGGGGCGAAACTCTGA	Y1H
OsPIL13-AD-F	TCAGAGGTGGACGCAAGCAATC	Full-length cDNA
OsPIL13-AD-R	CCAAAGCCAGAATAGCAGCAAC	Full-length cDNA

Supplementary Table 2. Detailed information of rice accessions with contrasting $iWUE_{FL}$ in Minicore rice population.

EFD	$iWUE_{FL}$ group	Latitude	Longitude	PLANTID	COUNTRY	Regions	New_Structure	PIL13 haplotypes
T4178	Low	46.2713	69.2312	Hi Muke	Kazakhstan	Balkans	AUS	Hap I
T4171	Low	32.0393	73.8355	P 35	India	Subcontinent	AUS	Hap I
P4134	Low	-34.4964	-58.2098	LA PLATA GENA F.A.	Argentina	South America	AUS	Hap I
T4175	Low	34.017	71.55	Daudzai Field Mix	Pakistan	Subcontinent	AUS	Hap I
S4166	Low	31.617	65.717	Spin Mere	Afghanistan	Subcontinent	AUS	Hap I
L4109	Low	25.75	93.25	ARC 6578	India	Subcontinent	AUS	Hap I
C4029	Low	24.9415	93.2684	IARI 6626	India	Subcontinent	AUS	Hap I
W4197	Low	26.7915	92.2167	ARC 10633	India	Subcontinent	IND	Hap I
R4151	Low	6.24301	81.427	Gallawa	Sri Lanka	Subcontinent	AUS	Hap I
S4161	Low	41.7316	70.2067	UZ ROS 7-13	Uzbekistan	Balkans	AUS	Hap I
W4194	High	17.0456	-76.9894	Sereno	Jamaica	Central America	IND	Hap II
E4045	High	1.97136	110.966	Acheh	Malaysia	South Pacific	IND	Hap II
P4138	High	23.2717	97.8197	KAUKKYI ANI	Myanmar	Southeast Asia	TRJ	Hap II
A4009	High	4.48964	157.9	WC 2811	Micronesia	South Pacific	TRJ	Hap II
Y4212	High	-14.5368	-45.1984	EMBRAPA 1200	Brazil	South America	TRJ	Hap II
B4013	High	13.0989	-90.6469	Secano do Brazil	El Salvador	Central America	TRJ	Hap II
L4103	High	38.42	-9.1	LUSITANO	Portugal	Western Europe	TEJ	Hap II
F4058	High	1.15745	23.707	Onu B	Zaire	Africa	TRJ	Hap II
K4098	High	22.6146	122.794	NANTON NO. 131	Taiwan	China	TRJ	Hap II
A4003	High	31.0886	-99.1713	E B Gopher	United States	North America	TRJ	Hap II

Supplementary Table 3. Frequency of major allele frequencies of SNPs surrounding *OsPIL13* based on low-coverage sequencing dataset in Minicore rice population.

Chromosome	SNP	Major Allele	Minor Allele	Major allele frequency
3	3m32430702	A	C	0.60
3	3m32431125	G	C	0.75
3	3m32431134	A	G	0.81
3	3m32431435	G	A	0.80
3	3m32431485	T	G	0.75
3	3m32431654	G	A	0.75
3	3m32432076	A	G	0.79
3	3m32432119	C	T	0.75
3	3m32432209	A	G	0.79
3	3m32432223	A	G	0.80
3	3m32433003	G	T	0.81
3	3m32433274	C	G	0.76
3	3m32433780	C	G	0.76
3	3m32434744	T	G	0.81
3	3m32435350	G	A	0.76

Supplementary Table 4. Haplotype analysis on allelic variation of nine SNPs at *OsPIL13* gene possessing significant association with $iWUE_{FL}$.

**Distance to ATG**	**Promoter region**						**CDS**		
	**-2017**	**-1716**	**-1075**	**-942**	**-928**	**-148**	**123**	**629**	**1593**
**SNP versions**	**v1**	**v2**	**v3**	**v4**	**v5**	**v6**	**v7**	**v8**	**v9**
***P*_value in GC**	**$1.87E^{-06}$**	**$1.87E^{-06}$**	**$1.87E^{-06}$**	**$1.87E^{-06}$**	**$1.87E^{-06}$**	**$1.87E^{-06}$**	**$1.87E^{-06}$**	**$1.87E^{-06}$**	**$1.87E^{-06}$**
Haplotype	3m32431134	3m32431435	3m32432076	3m32432209	3m32432223	3m32433003	3m32433274	3m32433780	3m32434744
Haplotype I (42 accessions)	A	G	A	A	A	G	C	C	T
Haplotype II (164 accessions)	G	A	G	G	G	T	G	G	G

Supplementary Table 5. Statistical analysis of the reads was performed based on transcriptome analysis.

Sample name	Raw reads	Clean reads	Mapped reads (%)	GC Content	%⩾Q30
WYG7_1	23557965	21793809	92.51	0.5239	0.945
WYG7_2	23961505	22146015	92.42	0.5281	0.933
WYG7_3	23555969	21773902	92.43	0.5259	0.944
PIL13^{v3m} _1	21170917	19561507	92.4	0.5316	0.935
PIL13^{v3m} _2	24563991	22755703	92.64	0.5306	0.945
PIL13^{v3m} _3	22439618	20774756	92.58	0.5263	0.949

Supplementary Figures

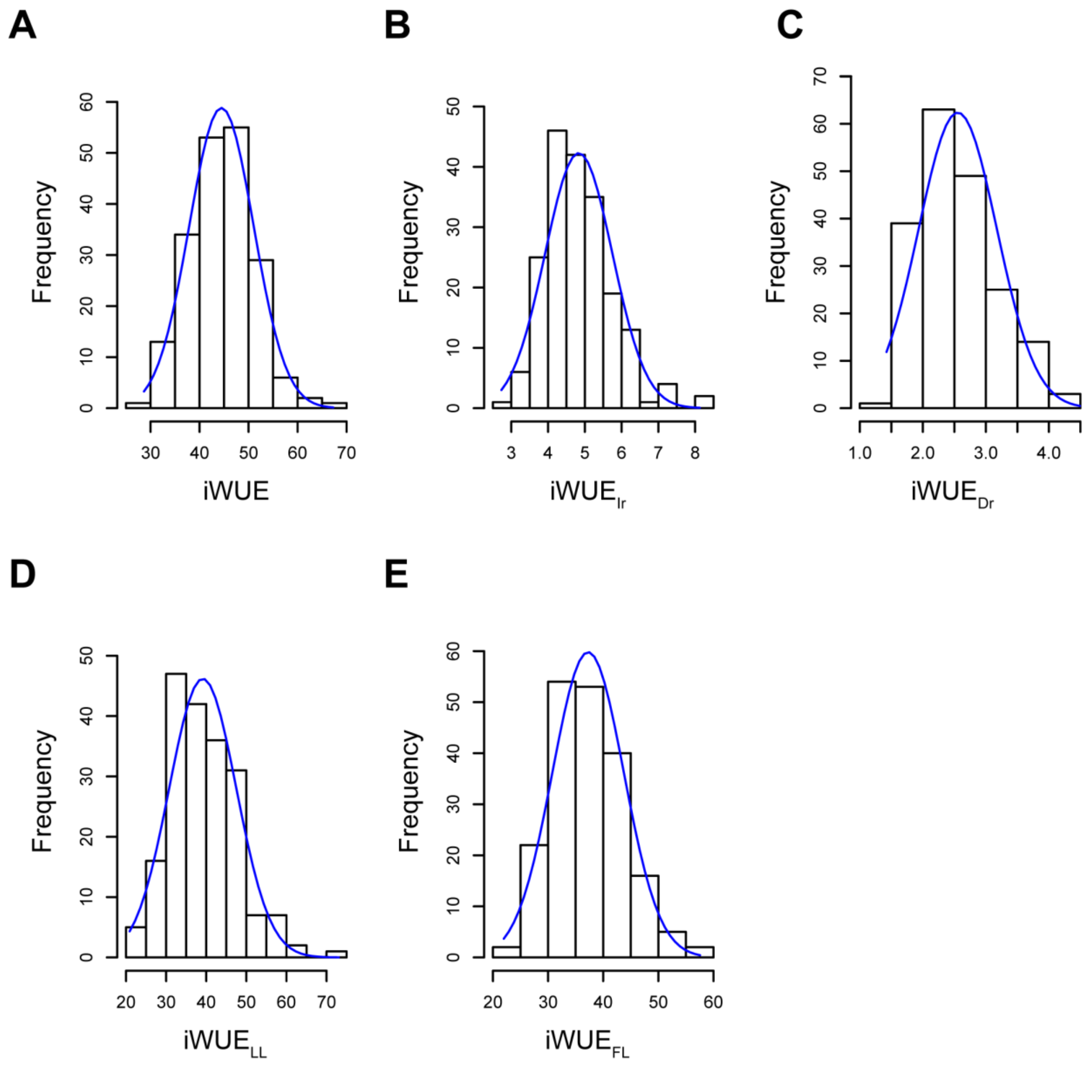

Supplementary Figure 1. Distribution of five iWUE parameters in the rice Minicore population exposed to FL-DS combined treatment in field. **A-E**, iWUE, $iWUE_{Ir}$, $iWUE_{Dr}$, $iWUE_{LL}$ and $iWUE_{FL}$. Details of combined FL-DS treatment and iWUE measurement were described in Material & Method section.

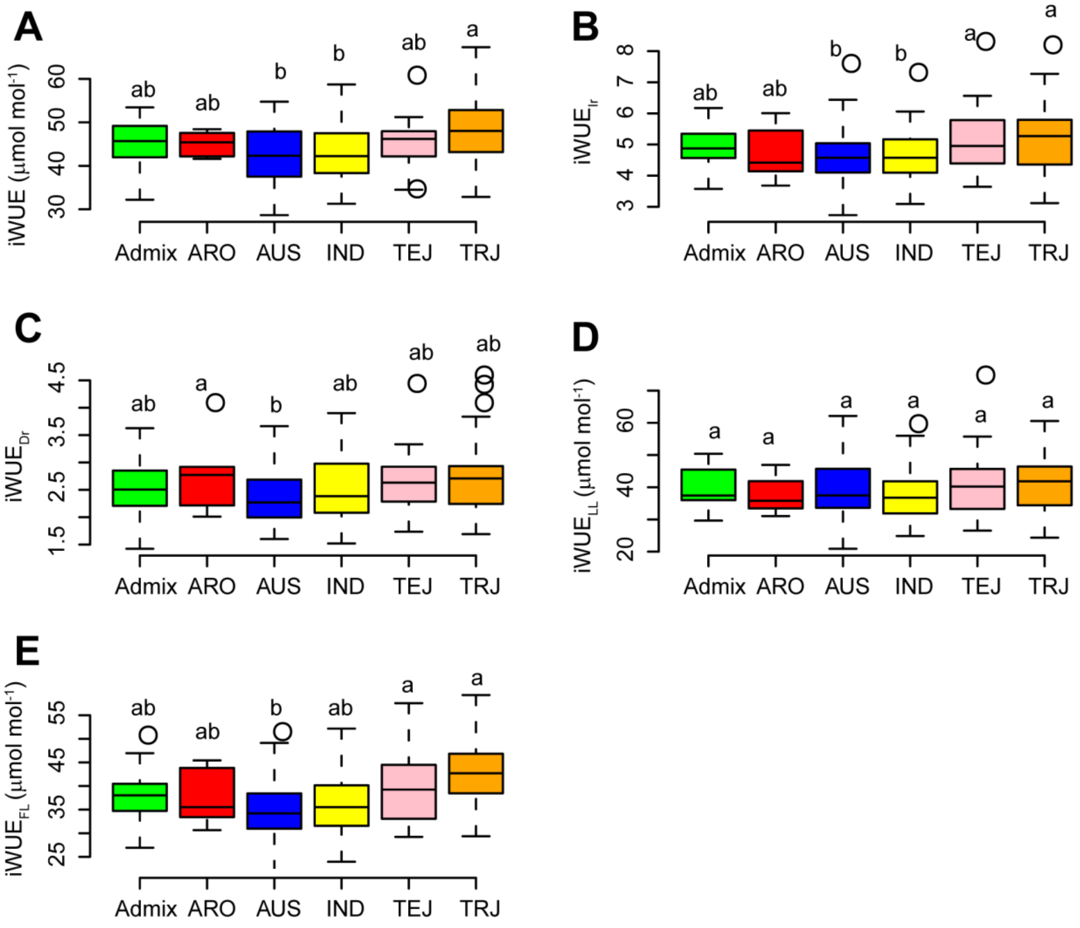

Supplementary Figure 2. Distribution of five iWUE parameters in the different subpopulations of the Minicore panel exposed to FL-DS treatment in field. **A-E**, iWUE. $iWUE_{Ir}$. $iWUE_{Dr}$. $iWUE_{LL}$ and $iWUE_{FL}$, respectively. In the boxplot, the edges represent the upper and lower quantiles, with the median value shown as a bold line in the middle of the box. The individual outside the range of the whiskers was shown as open dots. The Minicore population encompasses 19, 6, 38, 70, 30, and 37 accessions for Admix, ARO, AUS, IND, TEJ, and TRJ subpopulations, respectively. Minicore population consists of six subpopulations, i.e., ARO, AUS, IND, TEJ, TRJ, and admix, while *japonica* includes ARO, TEJ, and TRJ, and *indica* includes IND and AUS. Different letters represent the significant differences at a $P<0.05$ based on Tukey's HSD test ($P<0.05$).

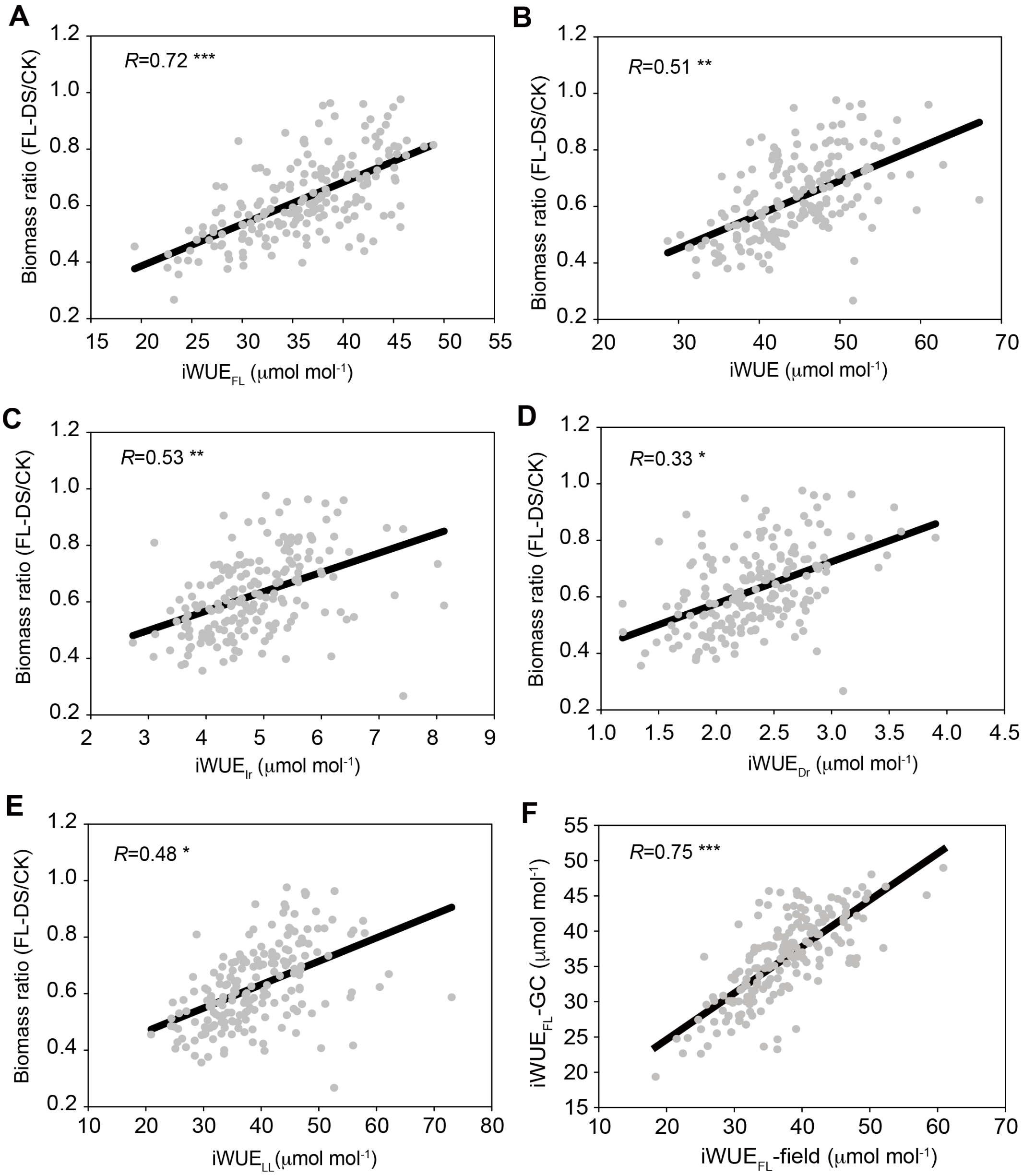

Supplementary Figure 3. Pearson correlation analysis among the five iWUE parameters and biomass ratio in FL-DS against CK condition. **A-E,** $iWUE_{FL}$, iWUE, $iWUE_{Ir}$, $iWUE_{Dr}$, and $iWUE_{LL}$. **F**, Pearson correlation between $iWUE_{FL}$ measured in the field and $iWUE_{FL}$ in GC. Pearson correlation coefficient (*R*) values were calculated, while asterisks represent the level of signifiance of *R* at $P<0.05$ (*), $P<0.01$ (**) and $P<0.001$ (***).

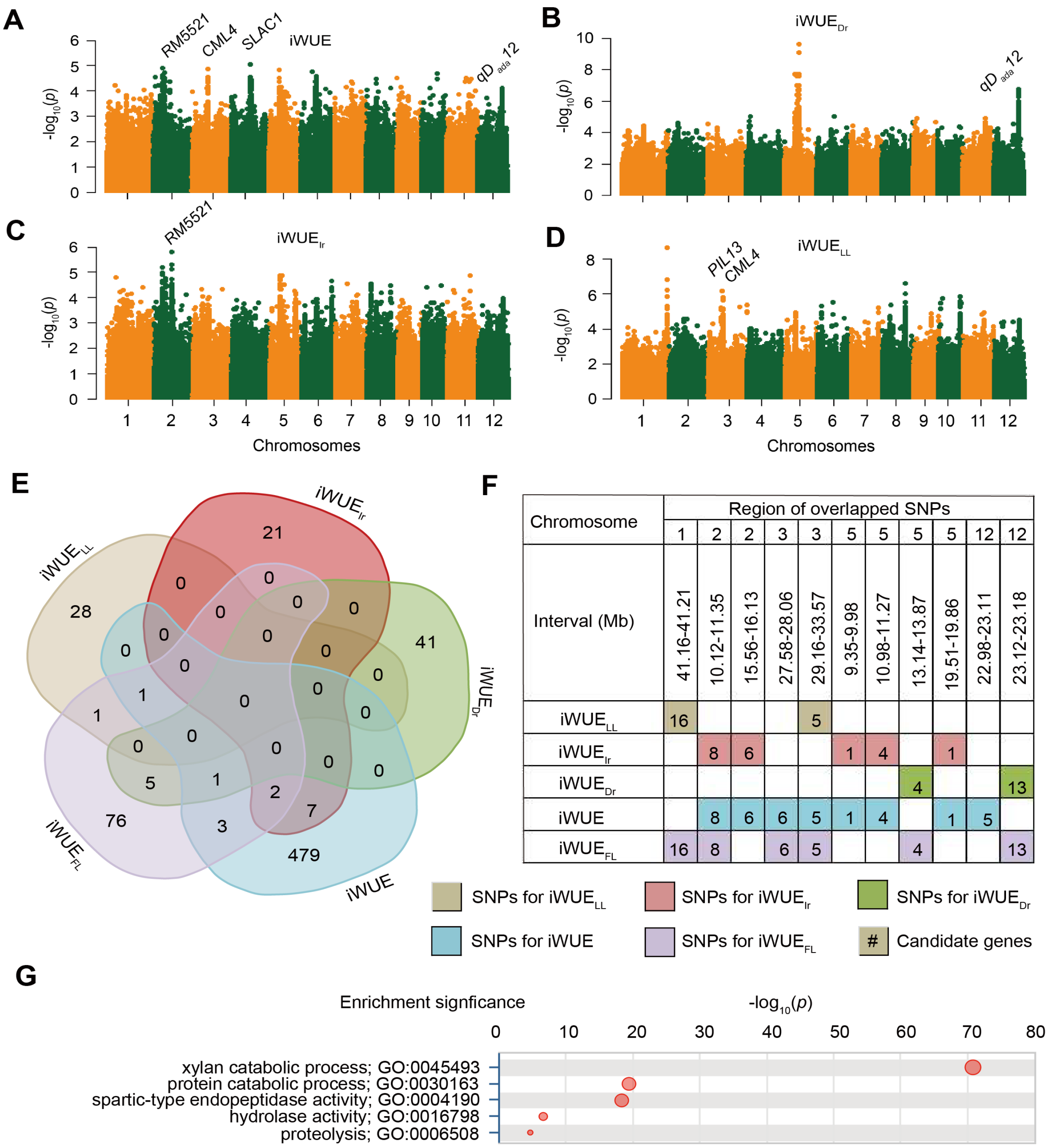

Supplementary Figure 4. Overlapped SNPs identified in different iWUE traits based on GWAS. **A-D**, Manhattan plots from the association mapping of the iWUE, $iWUE_{Dr}$, $iWUE_{Ir}$, and $iWUE_{LL}$. The reported QTLs-based genes were labeled in each panel, including *OsCML4*[10], *OsPIL13*[11], *RM5521*[12], *NAC6*[13], *SLAC1*[14] and *qD*$_{ada}$*12*[9]. **E**, Venn diagram representing the overlapped SNP significantly associated with five iWUE parameters. **F**, Distribution of overlapped SNPs associated considerably with five iWUE parameters. Different colors represent SNPs significantly associated with each trait. Numbers in cells represent the number of candidate genes corresponding to SNPs. **G**, Gene Ontology (GO) analysis on the list of candidate genes across five iWUE parameters.

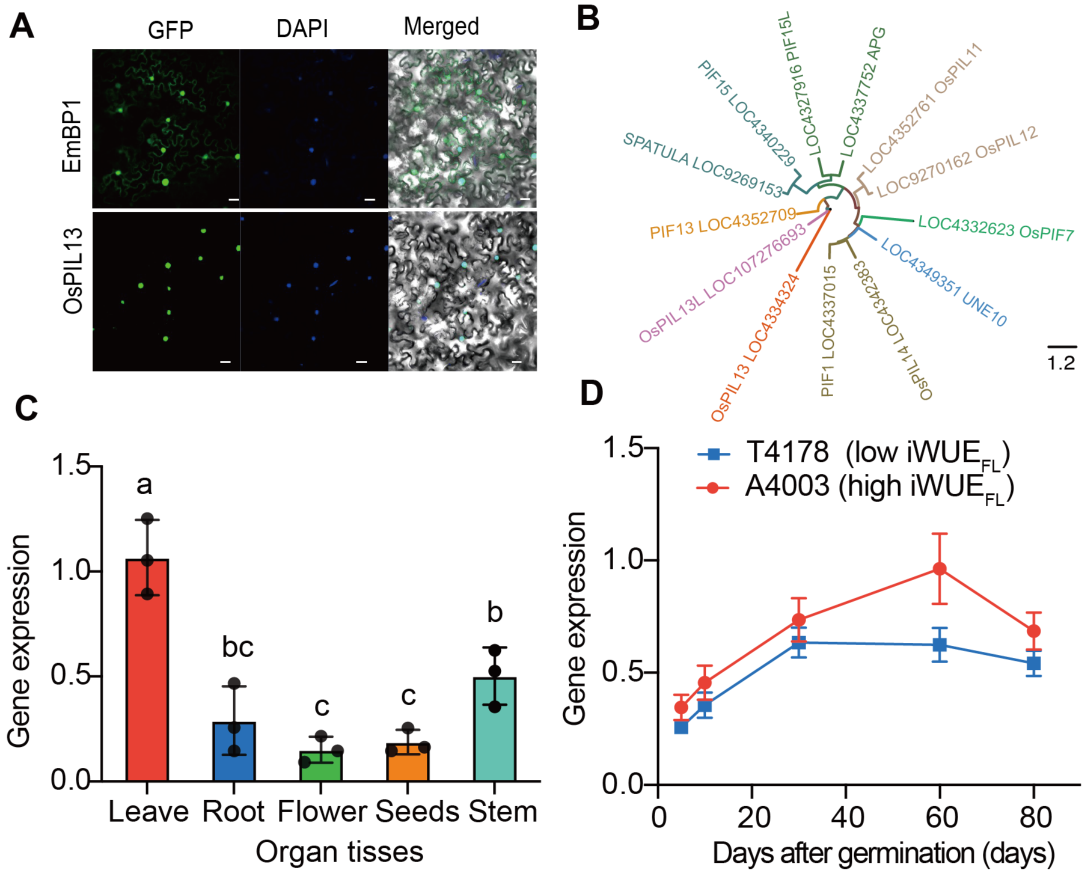

Supplementary Figure 5. Spatiotemporal analysis of *OsPIL13*. **A**, OsPIL13 protein located in nucleus. Subcellular localization was performed using transient transformation in tobacco leaves. A known bZIP transcription factor *EmBP1* gene[15] was used as a positive control. Bar, 100μm. **B**, Phylogenetic tree of *OsPIL13* and other 12 Phytochrome-interacting factors in rice genome based on protein alignment from NCBI database. **C**, *OsPIL13* gene expression among different organs. Different letters represent significant organ differences based on one-way *ANOVA* followed by Tukey's HSD tests. *n*=3. **D**, *OsPIL13* gene expression at different developmental stages within 80 days after germination (DAG) in the leaves between two rice accessions, i.e., A4003 belonging to high $iWUE_{FL}$ subgroups and T4178 belonging to low $iWUE_{FL}$ subgroups. *n*=3.

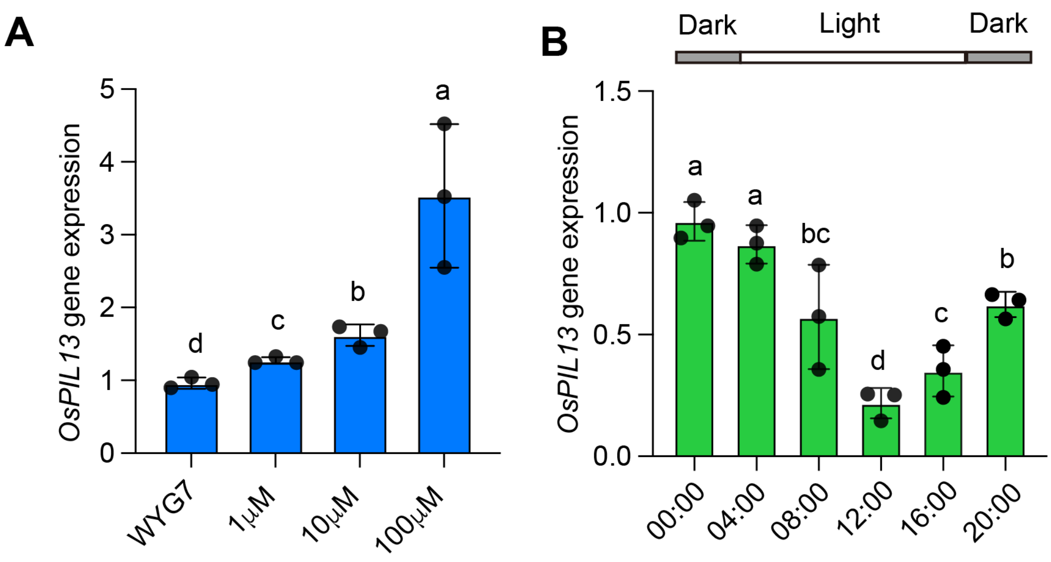

Supplementary Figure 6. Relative gene expression levels of *OsPIL13* in response to ABA and circadian rhythm patterns. **A**, ABA treatment. ~20 days WYG7 rice seedlings grown in GC under CK condition were sprayed by different ABA concentrations with 5 d interval for 10 d duration. **B**, Circadian rhythm. The leaves of ~20 days WYG7 rice seedlings grown in GC under CK condition were sampled at different time-points during the day. Different letters represent significant organ differences based on one-way *ANOVA* followed by Tukey's HSD tests. n=3.

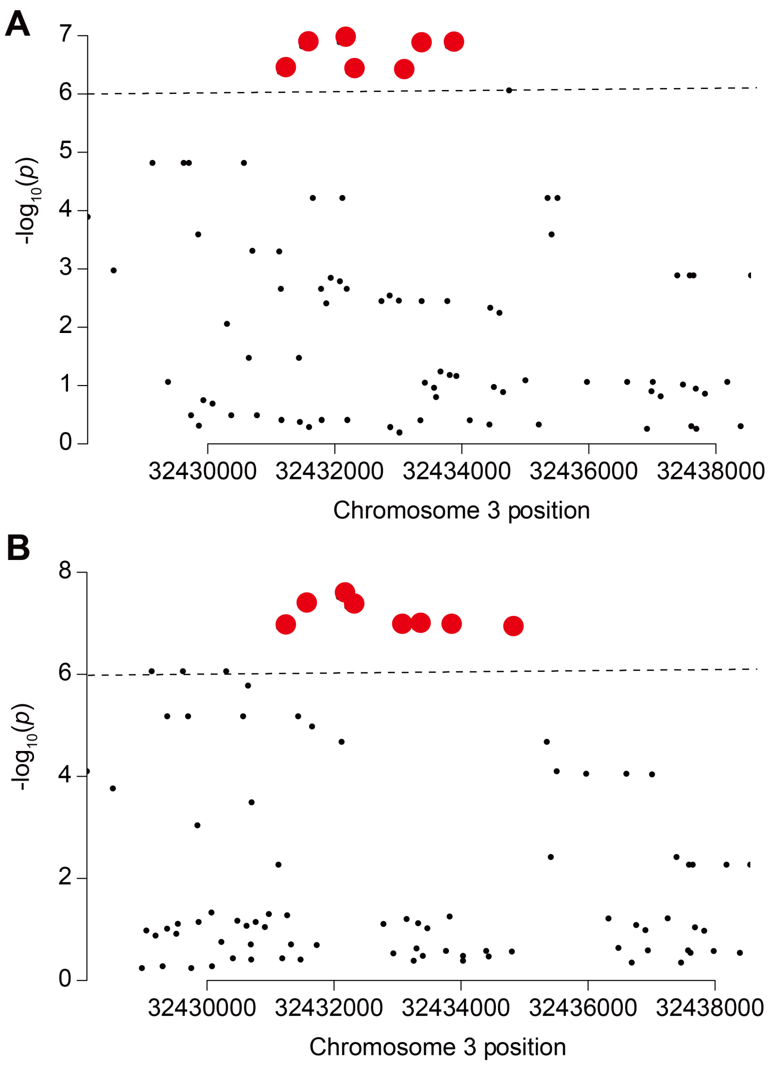

Supplementary Figure 7. The zoom-in Manhattan plot of nine SNPs at the *OsPIL13* gene associated with $iWUE_{FL}$ under both GC and field conditions. **A**, Field. **B,** GC condition. The nine SNPs were highlighted in red scatters.

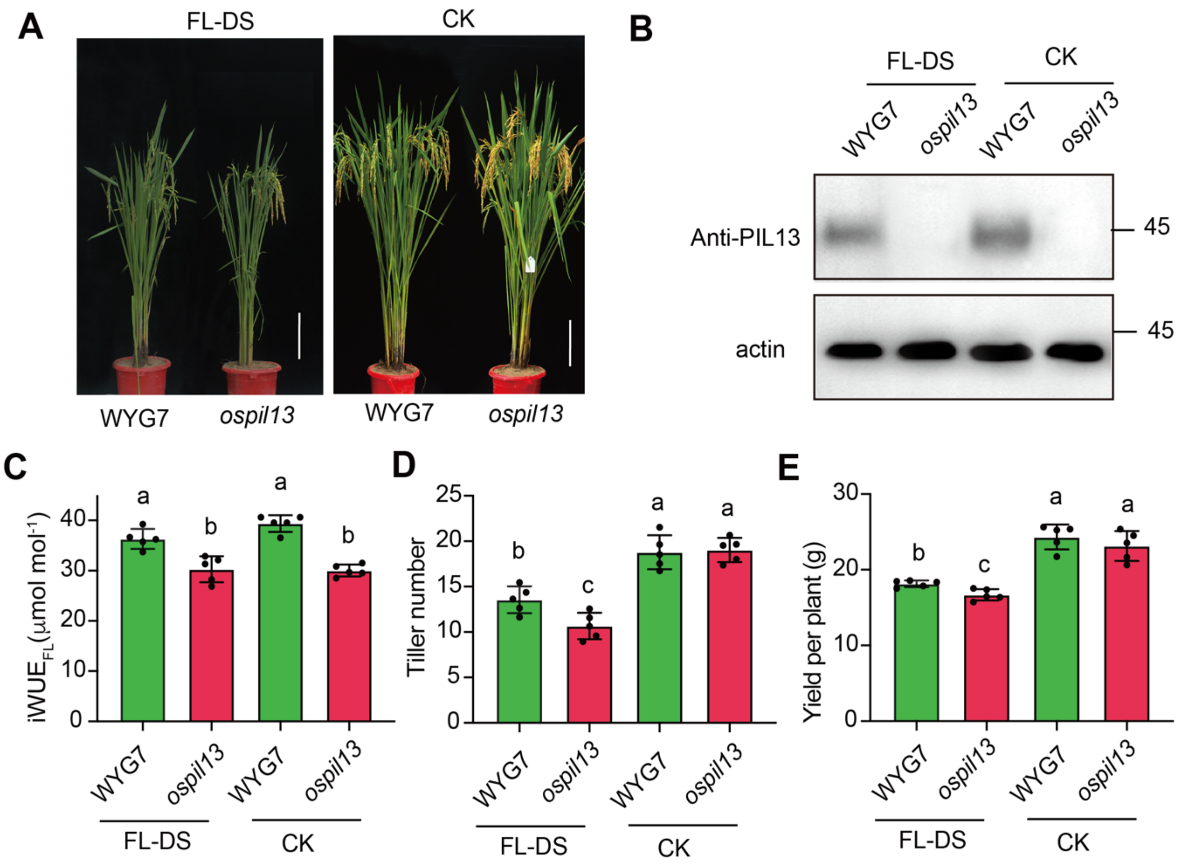

Supplementary Figure 8. Knocking-out *OsPIL13* leads to reduced iWUE$_{FL}$ under FL-DS. **A**, Performance of WYG7 and an *OsPIL13* mutant line (*ospil13*) exposed to either FL-DS or well-watered condition under HL (CK) in a GC at the graining stage. The vertical bar represents a scale of 10 cm. **B,** Western blotting results of protein expression of OsPIL13 in WYG7 and *ospil13* exposed to either FL-DS or CK condition. **C-E,** iWUE$_{FL}$, tiller number, and grain yield in WYG7 and *ospil13* exposed to either FL-DS or CK condition. Different letters represent significant differences among WYG7 and *ospil13* exposed to either FL-DS or CK condition based on one-way *ANOVA* followed by Tukey's HSD tests. For panel **B**, *n*=3 (individual plants) and for panel **C-E**, *n*=5 (individual plants).

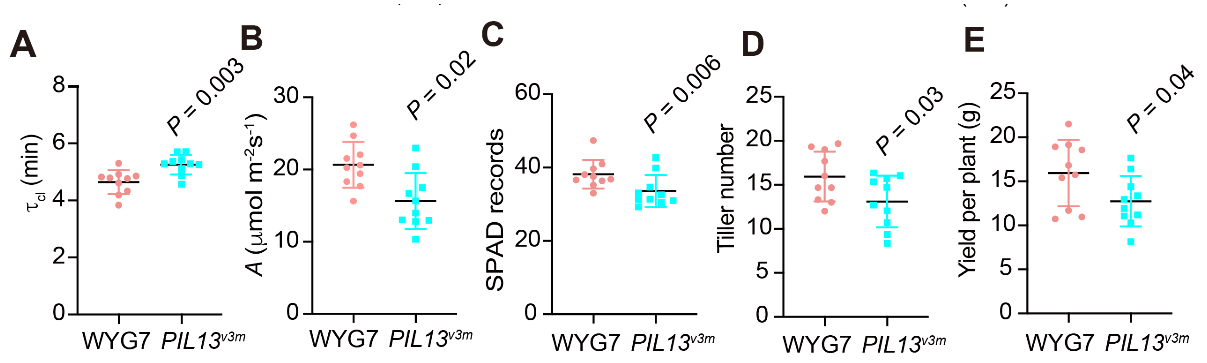

Supplementary Figure 9. Dynamics of A and g_s in WYG7 and *PIL13*^{v3m} exposed to a 20 d FL-DS treatment. **A-E,** τ_{cl}, A, chlorophyll contents (SPAD records), tiller number, and yield per plant in WYG7 and *PIL13*^{v3m} rice lines. A Student's t-test was implemented to determine the significance level between WYG7 and *PIL13*^{v3m} rice line for each trait. The parameter τ_{cl} represents the half-time (τ_{cl}) of stomatal closure speed[16]. The sample size is n=10 (individual plants).

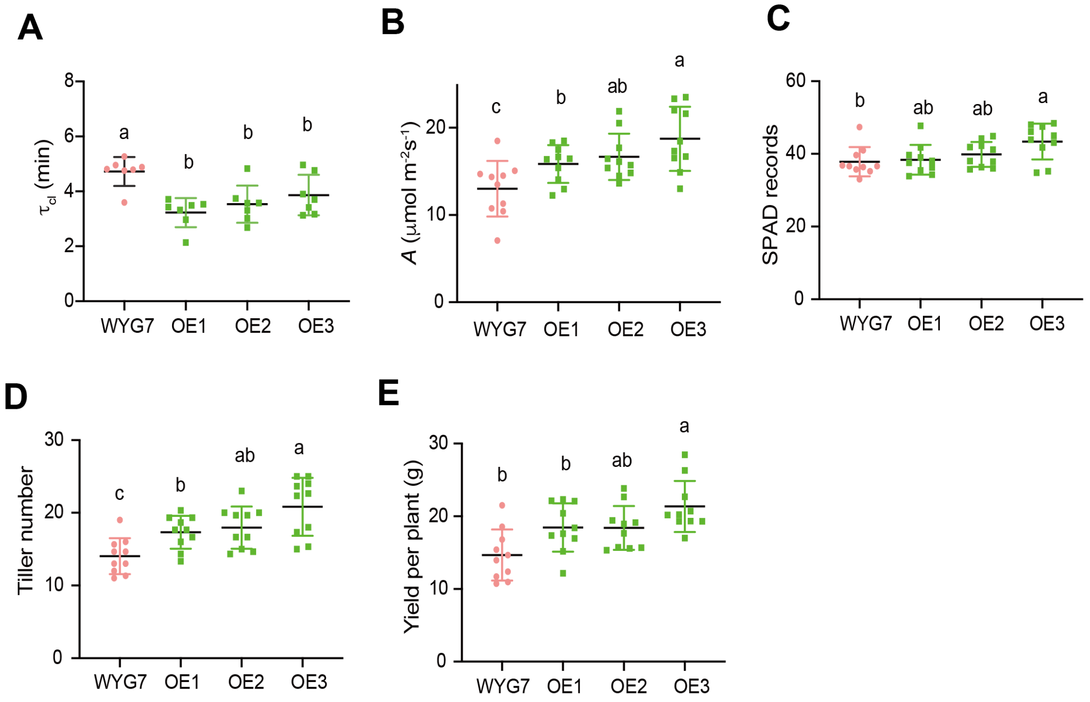

Supplementary Figure 10. Physiological and agronomic traits of *OsPIL13* overexpression lines under FL-DS condition. **A-E,** τ_{cl}, A, chlorophyll contents (SPAD records), tiller number, and yield per plant in WYG7 and three *OsPIL13* OE lines. Each bar data represents the mean of n replicates ±s.d.: n=7 (individual plants) for panel **A**, and n=10 for panels **B-E.** Different letters represent significant differences among WYG7 and three *OsPIL13*-OE lines based on

one-way *ANOVA* followed by Tukey's HSD tests.

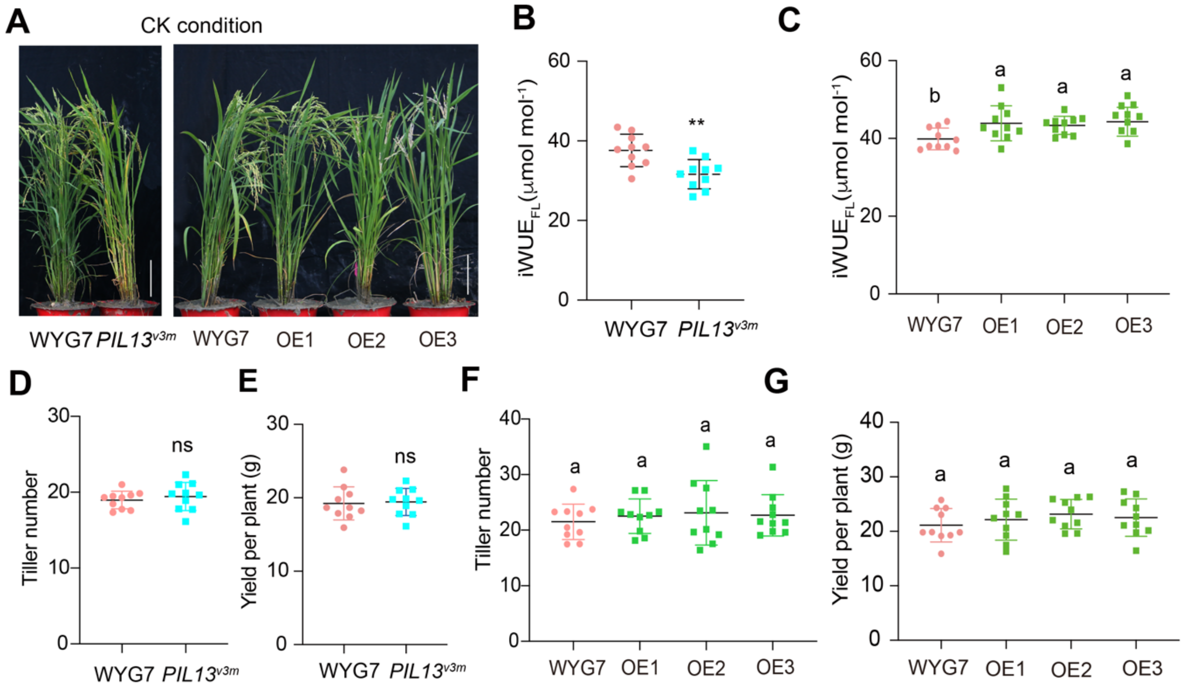

Supplementary Figure 11. Performance of *OsPIL13* transgenic rice lines under CK condition. **A**, Images of WYG7, *PIL13^{v3m}*, and *OsPIL13*-OE rice lines grown under CK condition at the graining stage. The vertical bar represents a scale of 10 cm. **B-C,** Comparisons of $iWUE_{FL}$ between WYG7 and *PIL13^{v3m}*, and among three *OsPIL13* OE lines. **D-E**, tiller number and yield per plant between WYG7 and *PIL13^{v3m}*. **F-G,** Comparisons of tiller number and yield per plant among WYG7 and three *OsPIL13* OE lines. For panels **B and D-E,** the Student's *t*-test was used to determine the significance level between WYG7 and *PIL13^{v3m}*. For panels **C, F-G,** different letters represent the significant differences among three *OsPIL13* OE lines and WYG7 based on Tukey's HSD test (P<0.05), n=10 (individual plants).

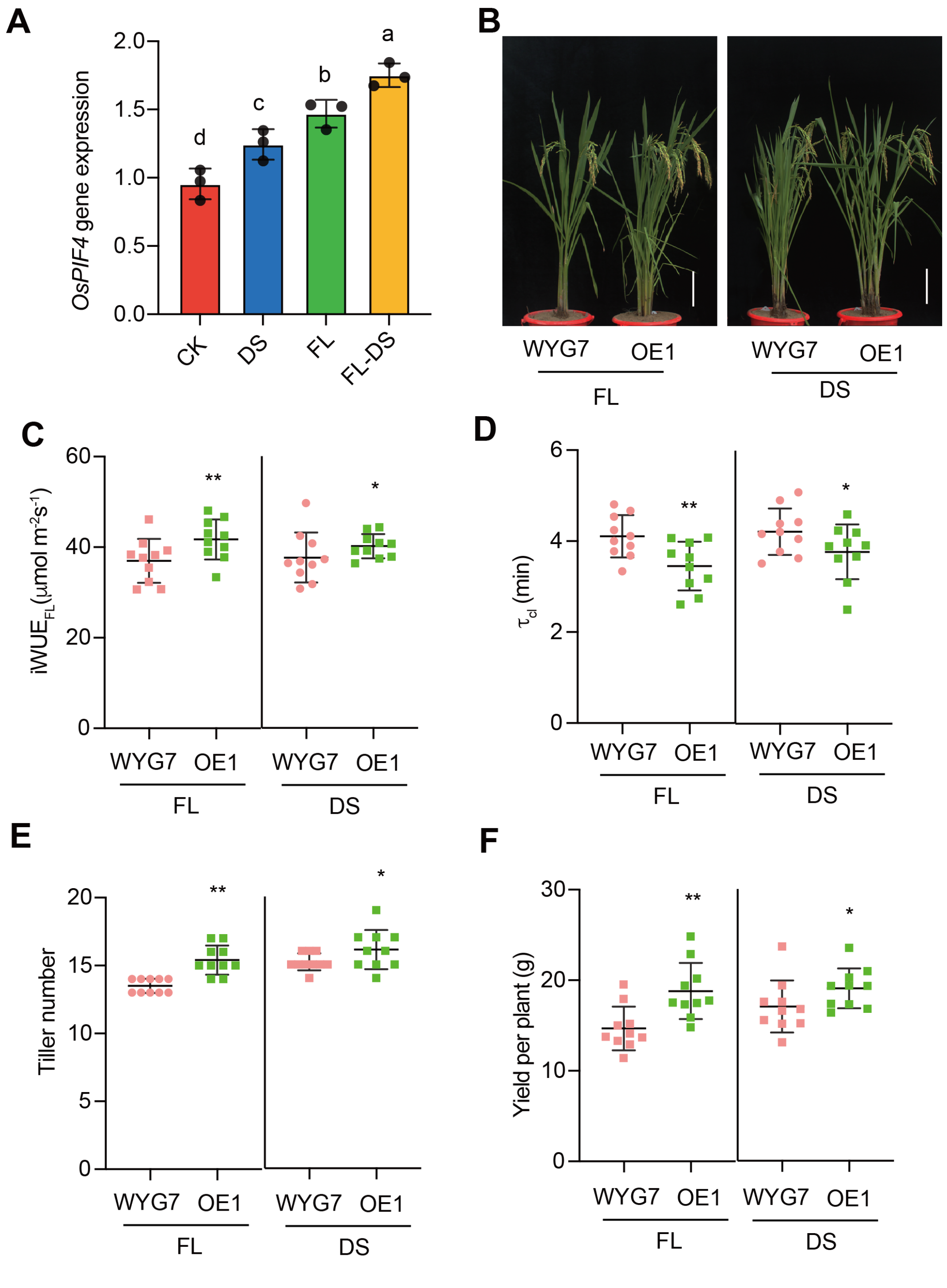

Supplementary Figure 12. Greater enhancement of iWUE$_{FL}$ by *Os*PIL13 under FL than under DS. **A,** Relative gene expression of *OsPIL13* in leaves of WYG7 exposed to four combinations of FL and DS for 20 d. The five treatments include: 1) 20 d CK (high light 1,500 µmol m^{-2}s^{-1} HL) without DS; 2) 20 d DS; 3) 20 d FL; 4) 10 d FL after 10 d DS; and 5) 20 d FL concomitant with DS. **B,** Images of WYG7 and an *OsPIL13* OE1 (OE1) line at the graining stage exposed

to either FL or DS for 20 d. The vertical bar represents a scale of 10 cm. **C-F,** iWUE$_{FL}$, τ_{cl}, tiller number and yield per plant between WYG7 and OE1 with either FL or DS treatment. Symbol "*" and "**" represent significant levels at *P*<0.05 and *P*<0.01, respectively, based on a Student *t*-test between WYG7 and OE1 for each trait. For panel **A**, *n*=3 (individual plants) and for panels **C-F**, *n*=10 (individual plants).

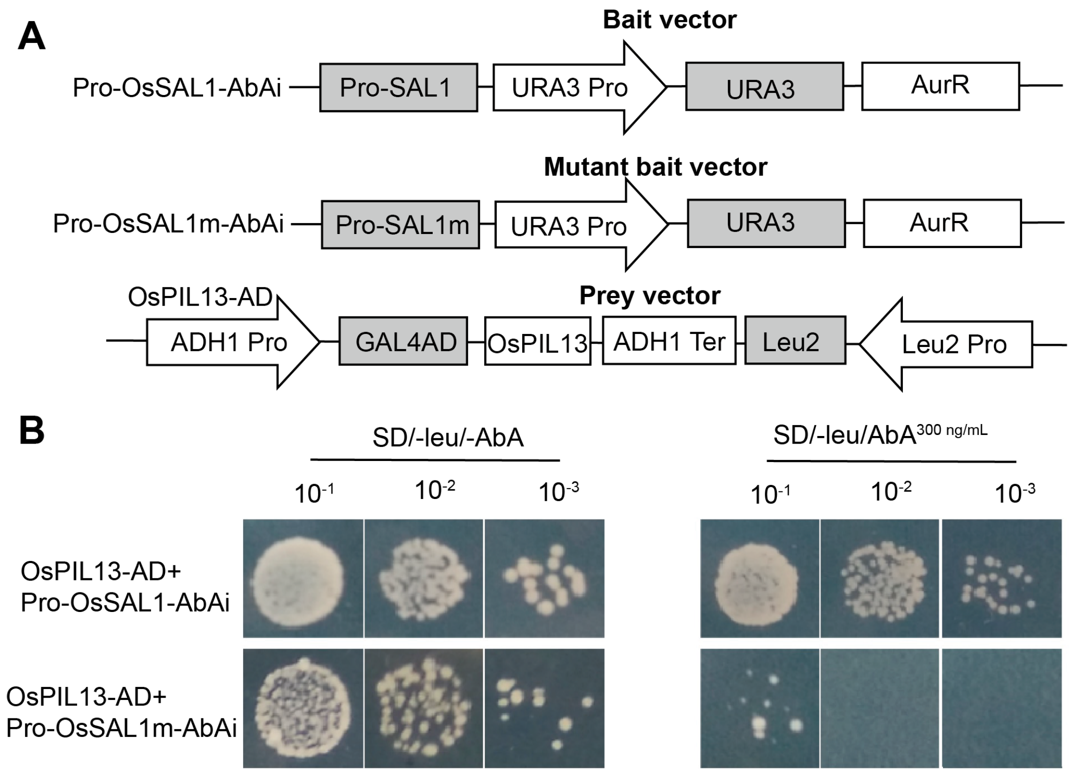

Supplementary Figure 13. *Os*PIL13 specifically binds to the G-box motif present in the *OsSAL1* promoter. **A**, Schematic diagrams of the promoter and mutant promoter fragments in *OsSAL1* used to construct the bait and mutant bait vectors. **B**, Y1H assays showing the interaction between *Os*PIL13 and CACGTG-motif present in the *OsSAL1* promoters, based on the ability of the transformed yeast strains to grow on SD/−Leu/AbA$^{300ng/mL}$ medium with gradient dilution (1/10, 1/100, 1/1,000). The transformants grown on SD/−Leu/−AbA plate were used as positive controls for the transformant's growth. Positive transformants were confirmed by spotting yeast cells onto an agar medium of SD/−Leu with 300 ng.ml^{-1} AbA. These assays were repeated three times with similar results.

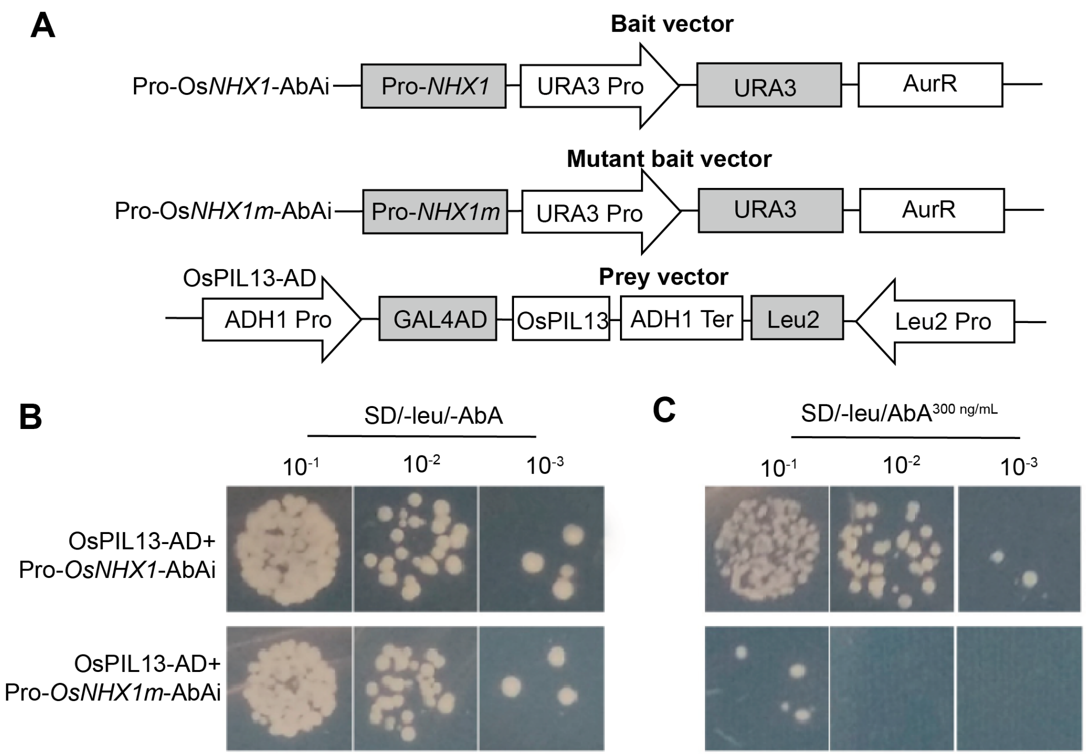

Supplementary Figure 14. *Os*PIL13 specifically binds to the G-box motif of the *OsNHX1* promoter. **A**, Schematic diagrams of the promoter and mutant promoter fragments in *OsNHX1* used to construct the bait and mutant bait vectors. **B-C**, Y1H assays show the interaction between *Os*PIL13 and the CACGTG motif in the *OsNHX1* promoters.

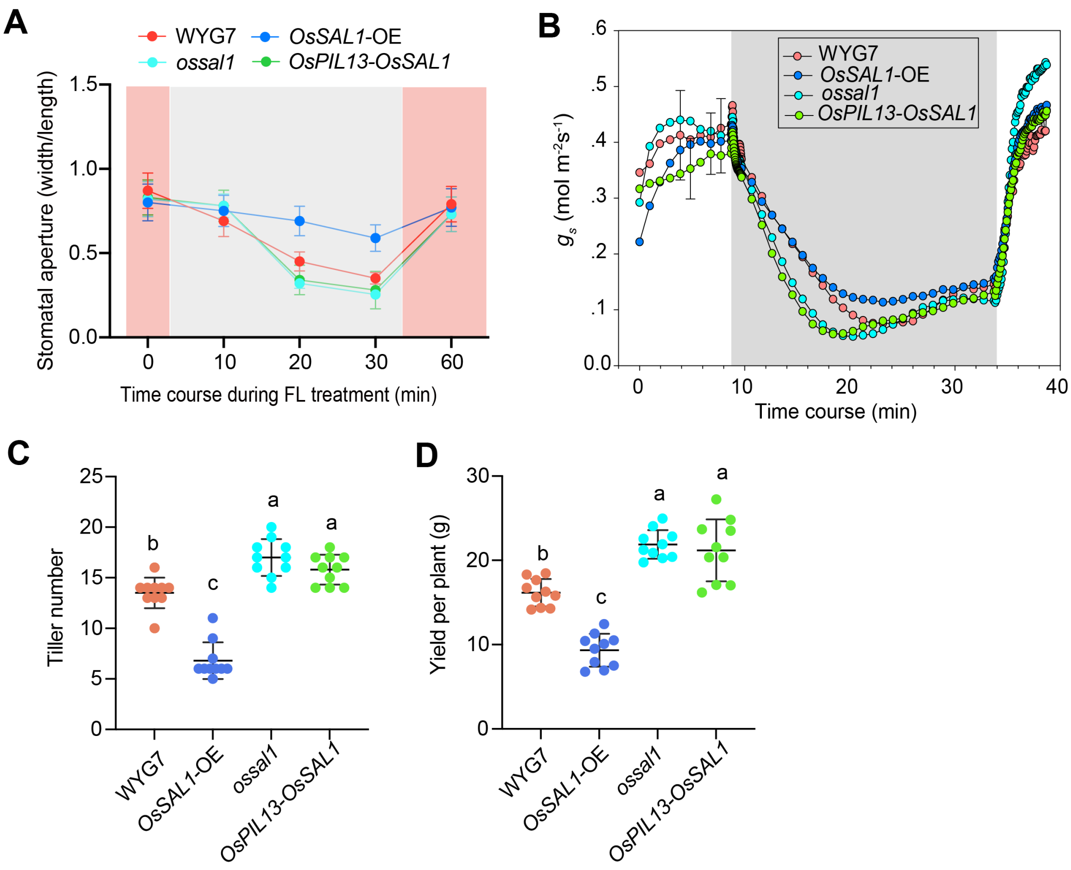

Supplementary Figure 15. Dynamics of *A* and g_s during FL-DS condition for WYG7 and *ossal1*, *OsSAL1*-OE, and a co-overexpression line of *OsPIL13* and *OsSAL1*. **A**, Stomatal aperture dynamic during FL. Data is referred to Fig. 6E. **B**, Stomatal conductances (g_s). **C-D,** Tiller number and yield per plant. For panel **A**, the vertical bar represents three independent replicate's maximum s.d. For panel **B**, each bar data represents the mean of n replicates (n=4, individual plants) ±s.d. For panels **C-D,** different letters represent significant differences among rice lines based on Tukey's HSD test (P<0.05), n=10 (individual plants).

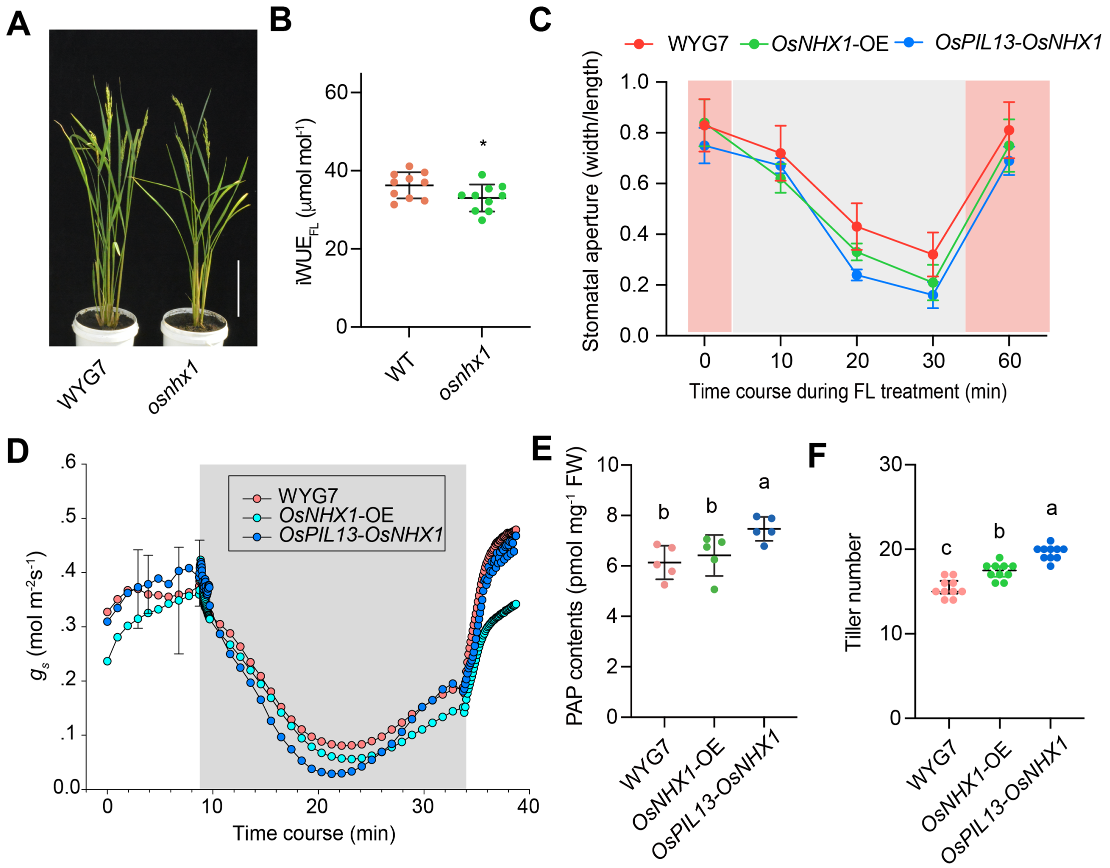

Supplementary Figure 16. Dynamics of *A* and g_s between WYG7, *OsNHX1*-OE, and a co-overexpression line of *OsPIL13-OsNHX1* under FL-DS condition. **A,** Performance of WYG7 and *osnhx1* exposed to 20 d FL-DS. The vertical bar represents a scle of 10 cm. **B,** iWUE$_{FL}$ between WYG7 and *osnhx1*. **C**, Stomatal aperture dynamics during FL. Data is referred to Fig. 6I. **D**, Stomatal conductance (g_s). **E-F,** Tiller number and PAP contents. For panel **B,** n=10. Symbol "*" represents P<0.05 based on a Student *t*-test. For panel **C**, the vertical bar represents three independent replicate's maximum s.d. For panel **D**, vertical bar represents the maximum s.d. from four independent replicates (n=4, individual plants) ±s.d. For panels **E-F,** different letters represent significant differences among rice lines based on Tukey's HSD test (P<0.05). For panel **E,** n=5 (individual plants) and for panel **F,** n=10 (individual plants).

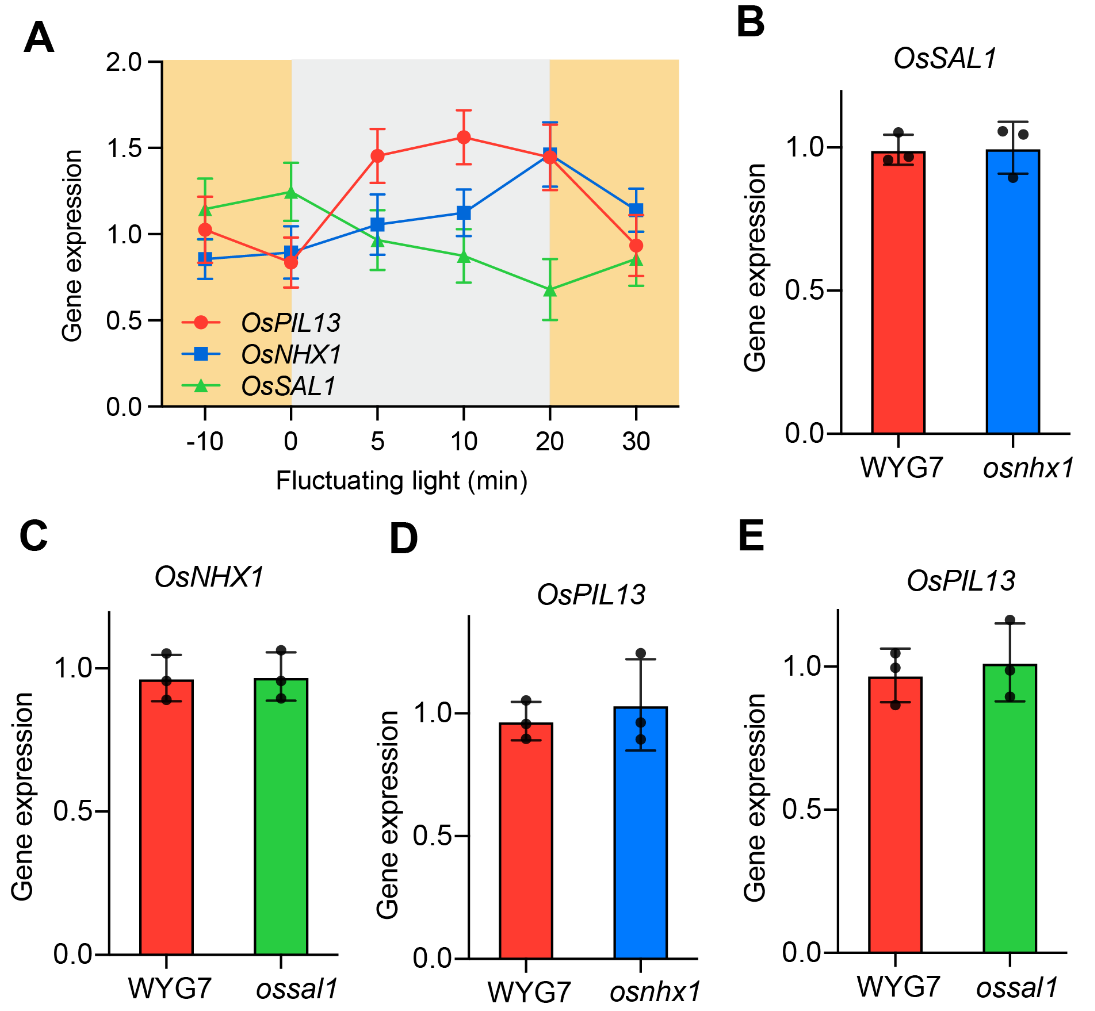

Supplementary Figure 17. Gene expression in mutants of *OsSAL1* and *OsNHX1* exposed to FL-DS. **A**, *in vivo* transcriptional dynamics of *OsPIL13*, *OsSAL1* and *OsNHX1* genes in WYG7 leaves during FL-DS. *OsSAL1* gene expression in *osnhx1* mutant. **B**, *OsSAL1*gene expression in *osnhx1*mutant. **C,** *OsNHX1* gene expression in *ossal1* mutant. **D-E,** *OsPIL13* gene expression in *osnhx1* (**D**) and *ossal1* mutant (**E**). Each bar data represents the mean of n replicates (*n*=3) ±s.d. For panels **B-E**, A Student's *t*-test was used to determine the significant levels between the mutants and WYG7 for each gene.

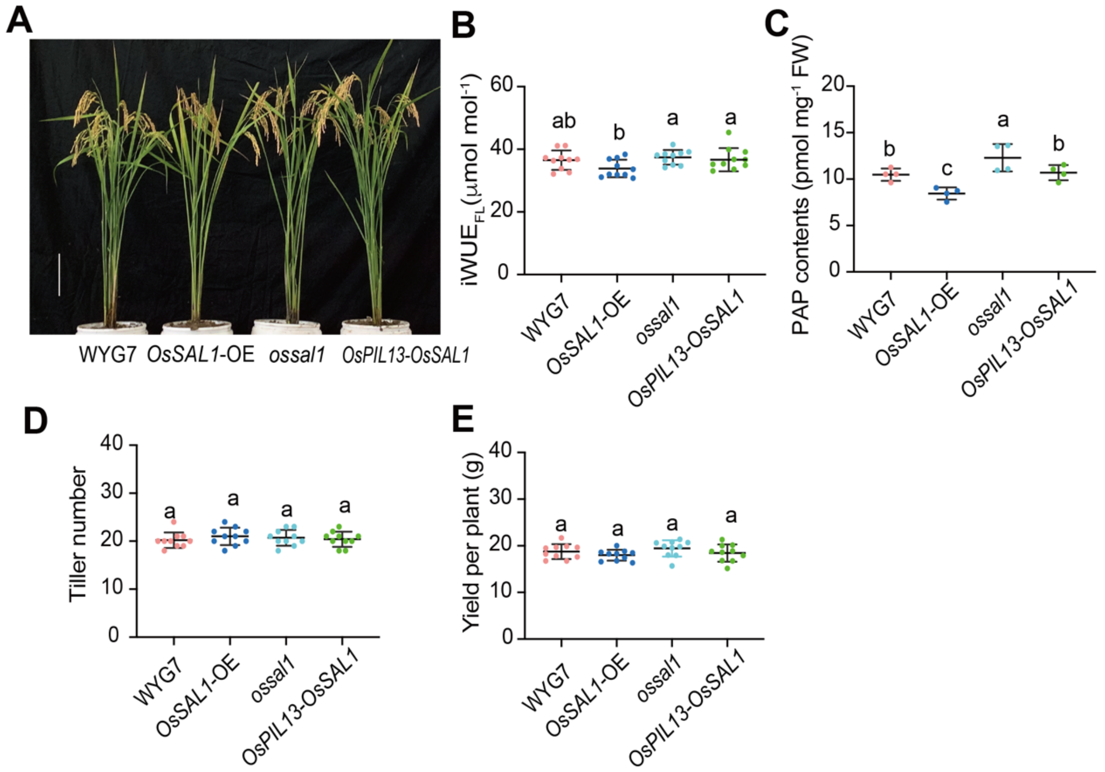

Supplementary Figure 18. The effects of *Os*PIL13 on *OsSAL1* in regulating iWUE under CK condition at the graining stage. **A**, Images of WYG7, *OsSAL1*-OE, *ossal1,* and co-overexpression of *OsPIL13* and *OsSAL1* rice lines exposed to CK condition. The vertical bar represents a scale of 10 cm. **B-E**, iWUE, PAP contents, tiller number, and yield per plant in WYG7, *OsSAL1*-OE, *osal1*, and a co-overexpression of *OsPIL13* and *OsSAL1* rice line (*OsPIL13-OsSAL1*). Each bar represents the mean of replicates (*n*=10 individual plants for panels **B, D-E**, and *n*=4 individual plants for panel **C**). Different letters represent significant differences among WYG7, *OsSAL1-OE*, *osal1,* and a *OsPIL13-OsSAL1* rice line based on Tukey's HSD test (P<0.05).

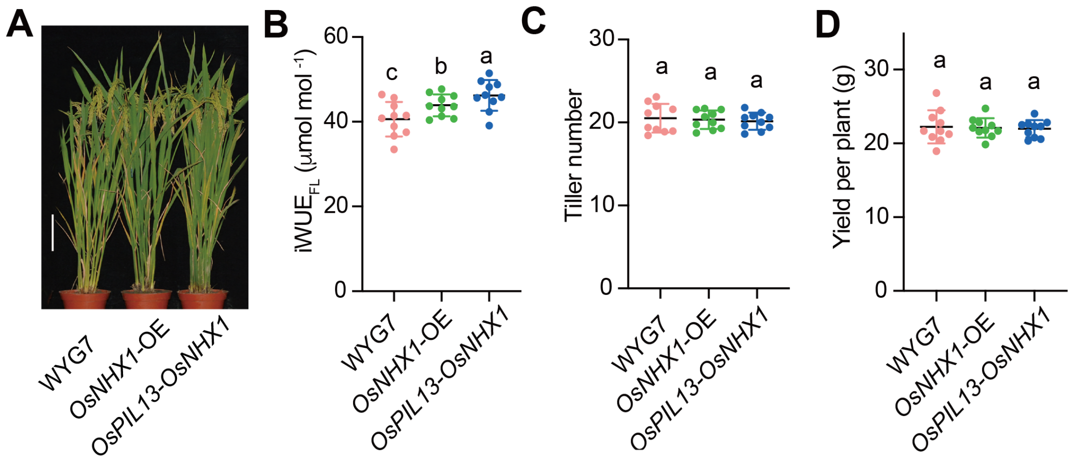

Supplementary Figure 19. Growth performance of WYG7, *OsNHX1*-OE, and a co-overexpression line of *OsPIL13* and *OsNHX1* under CK condition. **A**, Images of WYG7, *OsNHX1*-OE, and a co-overexpression rice line (*OsPIL13*- *OsNHX1*). The vertical bar represents a scale of 10 cm. **B**, Dynamics of iWUE during FL among WYG7, *OsNHX1*-OE, and *OsPIL13-OsNHX1* under CK condition. The vertical bar represents four independent replicates (*n*=4 individual plants) ±s.d. **C-D,** iWUE$_{FL}$, tiller number, and yield among WYG7, *OsNHX1*-OE, and *OsPIL13-OsNHX1* rice lines grown under CK condition. Each bar data represents the mean of replicates (*n*=10 individual plants) ±s.d. Different letters represent the significance level for each trait among WYG7, *OsNHX1*-OE, and *OsPIL13-OsNHX1* rice lines based on Tukey's HSD test (P<0.05).

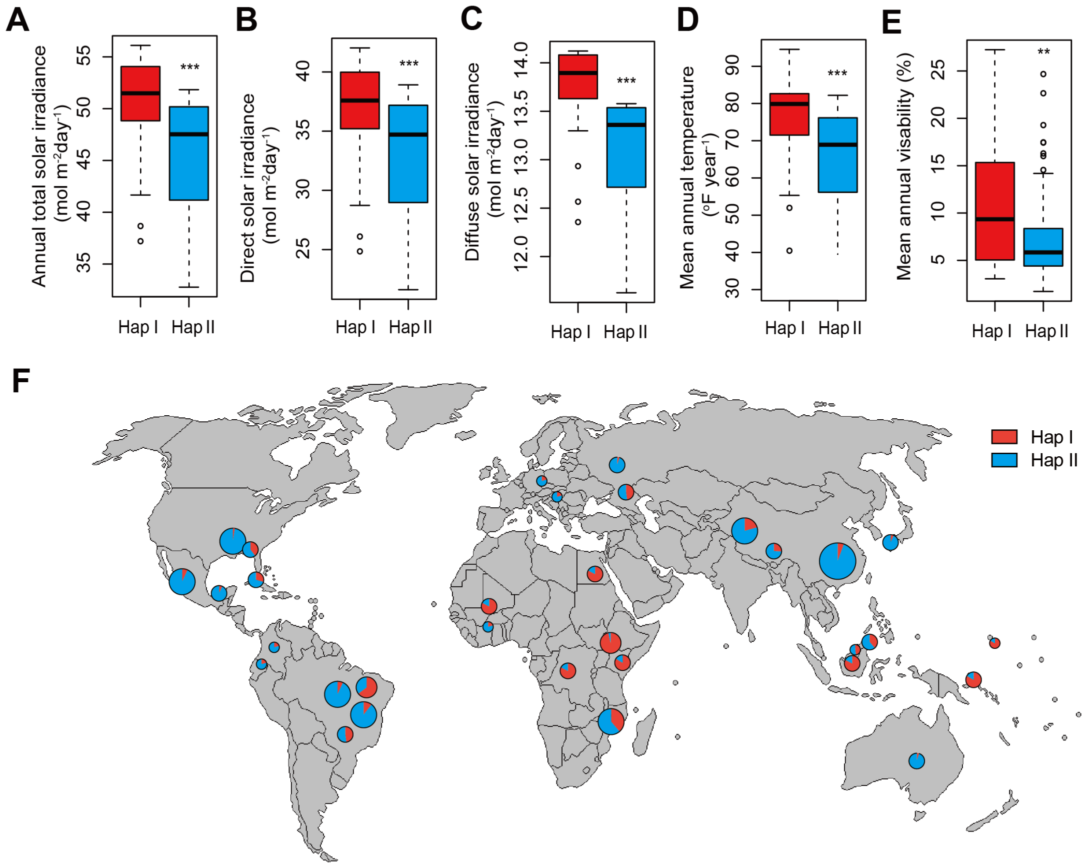

Supplementary Figure 20. Geographical distribution and original location climate of *OsPIL13* haplotypes in the Minicore population. **A-E**, Annual total irradiance, diffuse total solar irradiance, direct total solar irradiance, mean annual temperature, and mean annual visibility. **F,** Geographical distribution of *OsPIL13* haplotypes. The ratio of each haplotype to the total haplotypes was calculated for each country.

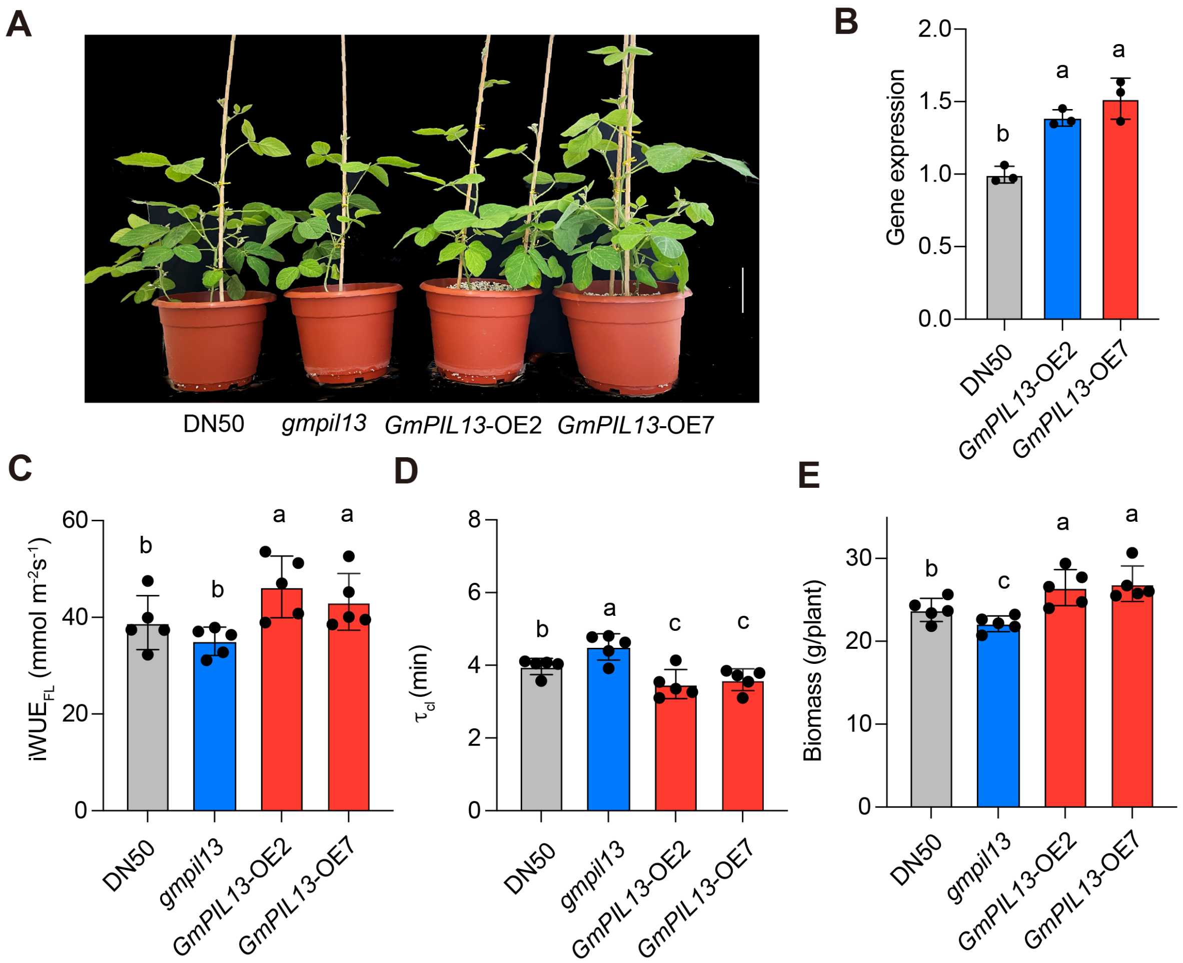

Supplementary Figure 21. *Gm*PIL13 promotes iWUE$_{FL}$ and biomass accumulation under CK condition in *Glycine max*. **A**, Performance of a *gmpil13* and two overexpression lines of *GmPIL13* in soybean with the background of Dongnong 50 (DN50) in CK (well-watered condition under HL) for 20 d. The vertical bar represents a scale of 10 cm. **B**, Gene expression levels of *GmPIL13* in two overexpression lines of *GmPIL13*. **C-E**, iWUE$_{FL}$, τ_{cl} and biomass in *gmpil1*3 and two overexpression lines of *GmPIL13* exposed to CK. Each bar data represents the mean of n replicates (*n*=3 individual plants for panel **B** and *n*=5 individual plants for panels **C-E**) ±s.d. Different letters represent the significance level for each trait among DN50, *gmpil1*3 and two overexpression lines of *GmPIL13* based on Tukey's HSD test (P<0.05).

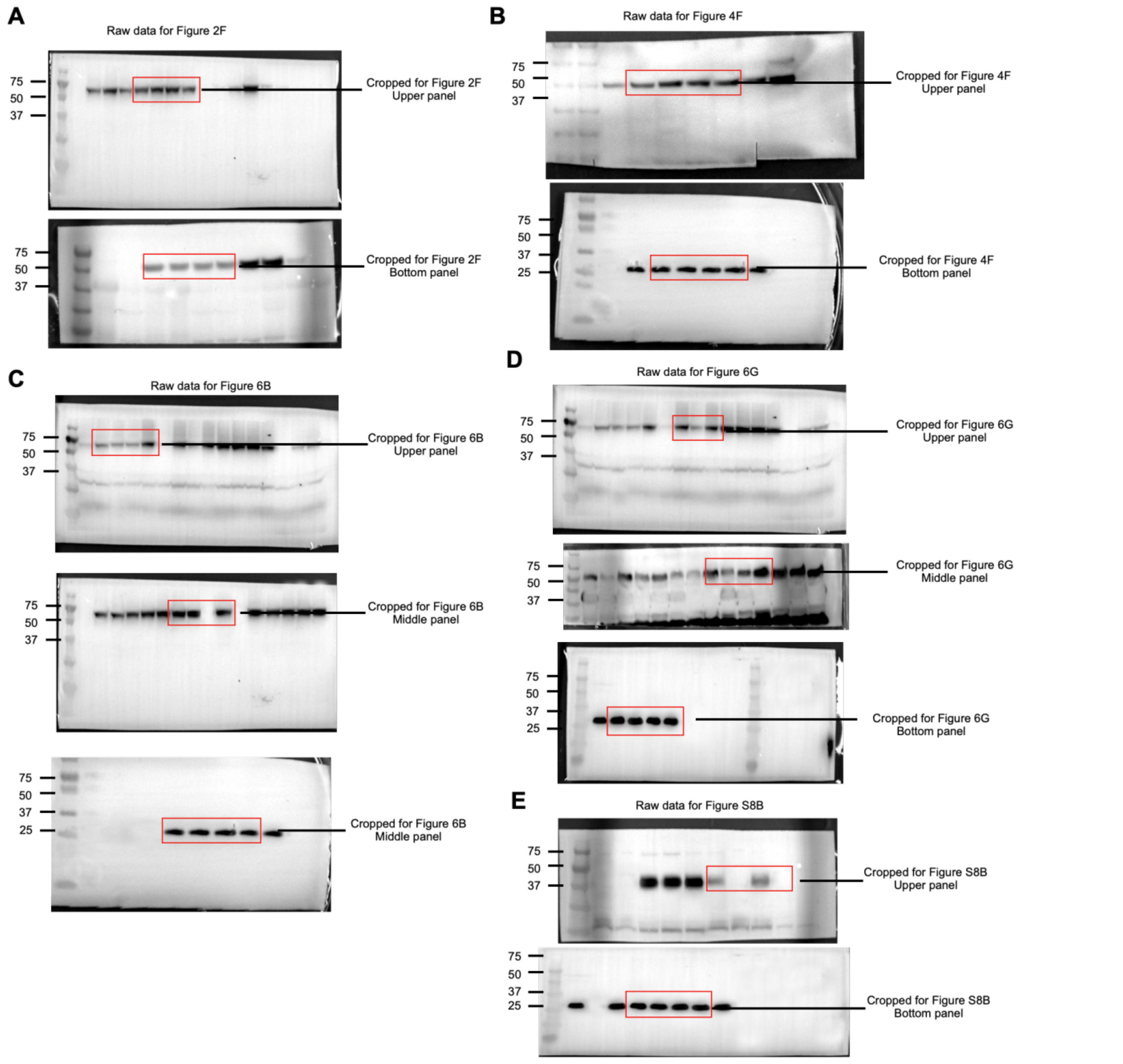

Supplementary Figure 22. Raw, uncropped data of gels for each data. Images were presented in the same order as in Figures: Fig. 2F (A), Fig. 4F(B), Fig. 6B(C), Fig. 6G(D) and Fig. S8B.

Supplementary references

1. Condon AG, Richards R, Rebetzke G, Farquhar G. Breeding for high water-use efficiency. *Journal of experimental botany* **55**, 2447-2460 (2004).
2. Wang H, *et al.* The power of inbreeding: NGS-based GWAS of rice reveals convergent evolution during rice domestication. *Molecular Plant* **9**, 975-985 (2016).
3. Qu M, *et al.* Rapid stomatal response to fluctuating light: an under-explored mechanism to improve drought tolerance in rice. *Functional Plant Biology* **43**, 727-738 (2016).

4. Qu M*, et al.* Alterations in stomatal response to fluctuating light increase biomass and yield of rice under drought conditions. *The Plant Journal* **104**, 1334-1347 (2020).
5. Wei Z, Duan F, Sun X, Song X, Zhou W. Leaf photosynthetic and anatomical insights into mechanisms of acclimation in rice in response to long-term fluctuating light. *Plant, Cell & Environment* **44**, 747-761 (2021).
6. Nishimura T, Nagao R, Noguchi T, Nield J, Sato F, Ifuku K. The N-terminal sequence of the extrinsic PsbP protein modulates the redox potential of Cyt b 559 in photosystem II. *Scientific reports* **6**, 21490 (2016).
7. Pereyra ME*, et al.* PIL13 enhances the expression of SAUR genes to promote growth in response to nitrate. *Proceedings of the National Academy of Sciences* **120**, e2304513120 (2023).
8. Zhao Y, Deng L, Last RL, Hua W, Liu J. Psb28 protein is indispensable for stable accumulation of PSII core complexes in Arabidopsis. *The Plant Journal*, (2024).
9. Chen H*, et al.* Genetic bases of the stomata-related traits revealed by a genome-wide association analysis in rice (Oryza sativa L.). *Frontiers in genetics* **11**, 611 (2020).
10. Yin XM, Huang LF, Zhang X, Wang ML, Xu GY, Xia XJ. OsCML4 improves drought tolerance through scavenging of reactive oxygen species in rice. *Journal of Plant Biology* **58**, 68-73 (2015).
11. Ma D*, et al.* Cryptochrome 1 interacts with PIL13 to regulate high temperature-mediated hypocotyl elongation in response to blue light. *Proceedings of the National Academy of Sciences* **113**, 224-229 (2016).
12. Adachi S*, et al.* Genetic architecture of leaf photosynthesis in rice revealed by different types of reciprocal mapping populations. *Journal of Experimental Botany* **70**, 5131-5144 (2019).
13. Nakashima K*, et al.* Functional analysis of a NAC-type transcription factor OsNAC6 involved in abiotic and biotic stress-responsive gene expression in rice. *The Plant Journal* **51**, 617-630 (2007).
14. Kusumi K, Hirotsuka S, Kumamaru T, Iba K. Increased leaf photosynthesis caused by elevated stomatal conductance in a rice mutant deficient in SLAC1, a guard cell anion channel protein. *Journal of experimental botany* **63**, 5635-5644 (2012).
15. Perveen S*, et al.* Overexpression of maize transcription factor mEmBP-1 increases photosynthesis, biomass, and yield in rice. *Journal of Experimental Botany* **71**, 4944-4957 (2020).
16. Vico G, Manzoni S, Palmroth S, Katul G. Effects of stomatal delays on the economics of leaf gas exchange under intermittent light regimes. *New Phytologist* **192**, 640-652 (2011).
