# Supplementary material for: The phytochrome-interacting factor PIL13 enhances water use efficiency under fluctuating light and drought resilience in rice and soybean
Source: Commun Biol. 2025 Aug 26;8:1286. doi: 10.1038/s42003-025-08605-8 (PMC12381223; doi:10.1038/s42003-025-08605-8)
Supplement: Supplementary file 2 — Description of Additional Supplementary Files [file 42003_2025_8605_MOESM2_ESM.docx]

**Description of Additional Supplementary Files**

**File name:** Supplementary Data 1
**Description:** The source data behind the graphs in different main figures (Figs. 1-7) in the paper.

**File name:** Supplementary Data 2
**Description:** The source data behind the graphs in different supplementary figures (Figures S1-S21) in the paper.

**File name:** Supplementary Data 3

**Description:** All variations of *OsPIL13* in 217 Minicore accessions identified by re-sequencing based on PCR amplification.
