# Supplementary material for: The phytochrome-interacting factor PIL13 enhances water use efficiency under fluctuating light and drought resilience in rice and soybean
Source: Commun Biol. 2025 Aug 26;8:1286. doi: 10.1038/s42003-025-08605-8 (PMC12381223; doi:10.1038/s42003-025-08605-8)
Supplement: Supplementary file 6 — Reporting-Summary [file 42003_2025_8605_MOESM6_ESM.pdf]

nature portfolio

Corresponding author(s): Mingnan Qu

Last updated by author(s): Jul 15, 2025

Reporting Summary

Nature Portfolio wishes to improve the reproducibility of the work that we publish. This form provides structure for consistency and transparency in reporting. For further information on Nature Portfolio policies, see our Editorial Policies and the Editorial Policy Checklist.

Statistics

For all statistical analyses, confirm that the following items are present in the figure legend, table legend, main text, or Methods section.

n/a	Confirmed	
☐	☒	The exact sample size (n) for each experimental group/condition, given as a discrete number and unit of measurement
☐	☒	A statement on whether measurements were taken from distinct samples or whether the same sample was measured repeatedly
☐	☒	The statistical test(s) used AND whether they are one- or two-sided<br>*Only common tests should be described solely by name; describe more complex techniques in the Methods section.*
☐	☒	A description of all covariates tested
☐	☒	A description of any assumptions or corrections, such as tests of normality and adjustment for multiple comparisons
☐	☒	A full description of the statistical parameters including central tendency (e.g. means) or other basic estimates (e.g. regression coefficient) AND variation (e.g. standard deviation) or associated estimates of uncertainty (e.g. confidence intervals)
☐	☒	For null hypothesis testing, the test statistic (e.g. F, t, r) with confidence intervals, effect sizes, degrees of freedom and P value noted<br>*Give P values as exact values whenever suitable.*
☒	☐	For Bayesian analysis, information on the choice of priors and Markov chain Monte Carlo settings
☒	☐	For hierarchical and complex designs, identification of the appropriate level for tests and full reporting of outcomes
☐	☒	Estimates of effect sizes (e.g. Cohen's d, Pearson's r), indicating how they were calculated

Our web collection on statistics for biologists contains articles on many of the points above.

Software and code

Policy information about availability of computer code

Data collection	metabolon; FLIR E40bx IR; ABI3730; LDcontroller FL053C.
Data analysis	MATLAB v.R2010a; GEMMA v.0.98.5; Haploview v.4.2; GCTA v.1.11.2; FastQC v 0.12.0; STAR 2.7.11b.

For manuscripts utilizing custom algorithms or software that are central to the research but not yet described in published literature, software must be made available to editors and reviewers. We strongly encourage code deposition in a community repository (e.g. GitHub). See the Nature Portfolio guidelines for submitting code & software for further information.

Data

Policy information about availability of data

All manuscripts must include a data availability statement. This statement should provide the following information, where applicable:

- Accession codes, unique identifiers, or web links for publicly available datasets
- A description of any restrictions on data availability
- For clinical datasets or third party data, please ensure that the statement adheres to our policy

The GEO number of RNA sequencing raw data is GSE284707. The detailed processed data of RNA sequencing were deposited in Supplementary Data 2. Supplementary Fig. 22 lists all Western blot experiments in the manuscript in uncropped and unedited forms. All source data are included in the “Supplementary data 1–2” file linked to this manuscript. The data used to substantiate the findings of this study are retrievable from the author at a reasonable request.

Research involving human participants, their data, or biological material

Policy information about studies with human participants or human data. See also policy information about sex, gender (identity/presentation), and sexual orientation and race, ethnicity and racism.

Reporting on sex and gender	not applicable
Reporting on race, ethnicity, or other socially relevant groupings	not applicable
Population characteristics	not applicable
Recruitment	not applicable
Ethics oversight	not applicable

Note that full information on the approval of the study protocol must also be provided in the manuscript.

Field-specific reporting

Please select the one below that is the best fit for your research. If you are not sure, read the appropriate sections before making your selection.

☒ Life sciences ☐ Behavioural & social sciences ☐ Ecological, evolutionary & environmental sciences

For a reference copy of the document with all sections, see nature.com/documents/nr-reporting-summary-flat.pdf

Life sciences study design

All studies must disclose on these points even when the disclosure is negative.

Sample size	Sample sizes were determined based on previous reports and best practices.
Data exclusions	No data were excluded from the analyses.
Replication	More than three replicates were taken for the measurements, all attempts at replication were successful.
Randomization	Due to the nature of the experimental setup blinding/randomisation was not practical.
Blinding	Due to the nature of the experimental setup blinding/randomisation was not practical.

Reporting for specific materials, systems and methods

We require information from authors about some types of materials, experimental systems and methods used in many studies. Here, indicate whether each material, system or method listed is relevant to your study. If you are not sure if a list item applies to your research, read the appropriate section before selecting a response.

Materials & experimental systems

n/a	Involved in the study	
☐	☒	Antibodies
☒	☐	Eukaryotic cell lines
☒	☐	Palaeontology and archaeology
☒	☐	Animals and other organisms
☒	☐	Clinical data
☒	☐	Dual use research of concern
☐	☒	Plants

Methods

n/a	Involved in the study	
☒	☐	ChIP-seq
☒	☐	Flow cytometry
☒	☐	MRI-based neuroimaging

Antibodies

Antibodies used	Anti-PIL13 (Agrisera; AS163955) Anti-NHX1 (Agrisera; AS09484); Anti-SAL1 (Agrisera; AS07256).
Validation	The antibodies were validated as provided in Agrisera website (https://www.agrisera.com/en/artiklar/pif4-phytochrome-interacting-

Validation	factor-4--2.html; https://www.agrisera.com/en/artiklar/nhe-1-na_h-antiporter-sodium_hydrogen-exchanger-.html; https://www.agrisera.com/en/artiklar/sal1.html

Plants

Seed stocks	The Rice Mini-Core Collection was requested from Prof. Chengcai Chu in South China Agricultural University. The population was obtained by USDA-ARS Genetic Stock Oryza Collection (http://www.ars.usda.gov/Main/Docs.htm?docid=23695)
Novel plant genotypes	A PIL13 mutant was produced via the CRISPR/CAS9 using a rice cultivar WYG7. This was accomplished using the CRISPR/CAS9 by the Biogle Company (Hangzhou, China). For the construction of the overexpression vector of PIL13, SAL1 and NHX1 genes, their cDNA sequences were amplified from WYG7, and were transferred into the pCAMBIA 1301 plasmid backbone carrying a GFP tag driven by their native promoters. Three homozygous OE lines with the highest expression of the PIL13 gene from the T3 generation were selected for drought and FL treatment experiments.
Authentication	The rice population was verified by Prof. Chengcai Chu in South China Agricultural University (https://pubmed.ncbi.nlm.nih.gov/27179918/).
